# Heterogeneity in the Frequency and Characteristics of Homologous Recombination in Pneumococcal Evolution

**DOI:** 10.1371/journal.pgen.1004300

**Published:** 2014-05-01

**Authors:** Rafal Mostowy, Nicholas J. Croucher, William P. Hanage, Simon R. Harris, Stephen Bentley, Christophe Fraser

**Affiliations:** 1 Department of Infectious Disease Epidemiology, Imperial College London, St Mary's Campus, London, United Kingdom; 2 Center for Communicable Disease Dynamics, Harvard School of Public Health, Boston, Massachusetts, United States of America; 3 The Wellcome Trust Sanger Institute, Wellcome Trust Genome Campus, Hinxton, Cambridge, United Kingdom; 4 Department of Medicine, University of Cambridge, Addenbrookes Hospital, Cambridge, United Kingdom; University of New Hampshire, United States of America

## Abstract

The bacterium *Streptococcus pneumoniae* (pneumococcus) is one of the most important human bacterial pathogens, and a leading cause of morbidity and mortality worldwide. The pneumococcus is also known for undergoing extensive homologous recombination via transformation with exogenous DNA. It has been shown that recombination has a major impact on the evolution of the pathogen, including acquisition of antibiotic resistance and serotype-switching. Nevertheless, the mechanism and the rates of recombination in an epidemiological context remain poorly understood. Here, we proposed several mathematical models to describe the rate and size of recombination in the evolutionary history of two very distinct pneumococcal lineages, PMEN1 and CC180. We found that, in both lineages, the process of homologous recombination was best described by a heterogeneous model of recombination with single, short, frequent replacements, which we call micro-recombinations, and rarer, multi-fragment, saltational replacements, which we call macro-recombinations. Macro-recombination was associated with major phenotypic changes, including serotype-switching events, and thus was a major driver of the diversification of the pathogen. We critically evaluate biological and epidemiological processes that could give rise to the micro-recombination and macro-recombination processes.

## Author Summary

*Streptococcus pneumoniae*, a bacterium commonly carried asymptomatically by children, is a major cause of diseases such as pneumonia and meningitis. The species is genetically diverse and is known to frequently undergo the remarkable process of transformation via homologous recombination. In this process, the bacterial cell incorporates DNA from other, closely related bacteria into its own genome, which can result in the development of antibiotic resistance or allow cells to evade vaccines. Therefore it is important to quantify the impact of this process on the evolution of *S. pneumoniae* to understand how quickly the species can respond to the introduction of such clinical interventions. In this study we followed the recombination process by studying the evolution of two important and very different lineages of *S. pneumoniae*, PMEN1 and CC180, using newly available population genomic data. We found that pneumococcus evolves via two distinct processes that we term micro- and macro-recombination. Micro-recombination led to acquisition of single, short DNA fragments, while macro-recombination tended to incorporate multiple, long DNA fragments. Interestingly, macro-recombination was associated with major phenotypic changes. We argue that greater insight into the adaptive role of recombination in pneumococcus requires a good understanding of both rates of homologous recombination and population dynamics of the bacterium in natural populations.

## Introduction

The evolution of many bacterial species is largely driven by horizontal exchange of sequence. Often, this can be attributed to the movement of autonomously mobile genetic elements (MGEs). Many of those are able to insert into the host chromosome through site-specific recombination mediated by an integrase. However, in ‘naturally’ transformable species that possess a competence system, exogenous DNA can be imported from the environment and integrated into the chromosome through homologous recombination (HR). This process was first discovered in *Streptococcus pneumoniae* (the pneumococcus), representing some of the earliest work on molecular genetics [1]. Initially, recombination was considered by many microbiologists to be interesting but rare. However, later population-based studies demonstrated that it can have a quantifiable impact on population genetic structure of many bacteria, including *S. pneumoniae* [2]–[4]. Additionally, as this mechanism only requires that the acquired DNA is homologous at the ends, recombination allows for the cassette-like transfer of highly variable genes, such as those that encode for the pneumococcal capsule [5], [6], in a process originally defined as ‘homology-directed illegitimate recombination’ [7]. This has important clinical consequences, as this exchange of sequence has played a crucial role in the development of pneumococcal antibiotic resistance [8], as well as the ‘switching’ of capsule types that can result in vaccine escape [9], [10].

The rate at which the recombination process occurs is of importance when considering the adaptation of the bacterium to clinical interventions. The simplest null expectation is that HR is a homogeneous process across the species. However, recent findings suggest that homogeneity of recombination is unlikely to capture the dynamics of horizontal sequence exchange in pneumococci. In particular, heterogeneity has been observed in the rates at which different genotypes accumulate sequence diversity through HR. Analysis of multilocus sequence typing data identified a subset of ‘hyper-recombinant’ pneumococci that were more likely to be resistant to a number of antibiotics [11]; similarly, comparison of lineages within a single population found significant variation in the observed rate of HR [12]. Second, *in vitro* work has found that the frequency of recombination events occurring across the genome in isogenic recipient bacteria varies with the concentration of donor DNA, suggesting the environment is likely to influence the process of sequence transfer [13]. Similarly, extensive exchanges between pneumococci over short time periods have also been observed in clinical isolates, sometimes with important phenotypic consequences [14]–[16]. Third, variation has been observed in the rate at which pneumococci undergo transformation in experimental systems [17], [18]. Therefore more detailed quantification of the observed contribution of HR will be invaluable in defining and understanding the behaviour of distinct lineages under different conditions. This in turn should help us understand how recombination contributes to the overall rate of diversification, and how it drives adaptive changes in pneumococcal populations.

The opportunity for such an analysis is presented by the recent whole genome sequencing of two international collections representing contrasting pneumococcal genotypes. The first is a set of 241 pneumococcal genomes of the recently emerged pandemic multidrug resistant lineage, PMEN1 [19]. This lineage appears to have originated in Europe in 1970s, and in the following decades spread rapidly across the world. The ancestral serotype of this lineage, serotype 23F, has switched to new capsules by HR which have resulted in its evasion of the 7-valent vaccine introduced in the early 2000s. The second lineage is a set of serotype 3 isolates belonging to clonal complex 180 (CC180) [20]. Serotype 3, which causes disease associated with high levels of mortality, has been recently included in the expanded 13-valent conjugate vaccine formulation. The CC180 lineage appears to be older than PMEN1, yet there is little evidence of it having undergone homologous recombination in recent decades, with the consequence that it is generally susceptible to antibiotics and has not altered its serotype. Hence these two genotypes, PMEN1 and CC180, are highly distinct both in terms of their phenotypes and evolutionary dynamics.

This work describes the fitting of different mathematical models of sequence exchange to the HR identified in the PMEN1 and CC180 datasets in order to identify and characterise and heterogeneity evident in the process. This resulted in the identification of two different classes of HR in both lineages: micro-recombination and macro-recombination. Potential underlying mechanistic explanations for this observation, and the implications for bacterial evolution, are discussed.

## Methods

In this section we give a short summary of the methods used here, including the datasets used, the approach and mathematical models. The full description, including the notation used and the derivation of the models, is given in supplementary Text S1.

### Structure of the data

The analysis presented here is based on the inference of individual HR events, as previously described by Croucher et al. [19]. Briefly, this approach identifies independent HR events as clusters of SNPs in a genealogy reconstructed from whole genome alignments. Removal of those events allows to establish a clonal tree based on vertical transmission of SNPs. The inference for the PMEN1 lineage was based on an alignment of 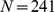 sequences, resulting in a genealogy with 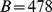 branches and 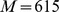 homologous recombinations, whereas the inference for the CC180 lineage was based on an alignment of 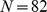 sequences, resulting in a genealogy with 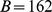 branches and 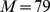 homologous recombinations.

Let 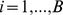 label the branches, and let 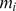 be the number of HR events assigned to branch 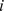, such that 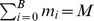. For a given branch 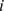, let 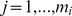 label the recombination events, and let 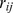 be the length of genetic tract, in DNA base pairs, replaced by the HR event. We define the recombination rates in our models as rates per unit of branch length. Thus, their interpretation depends on the chosen measure of branch length. Since our model structure is generic with respect to this choice, by default the branch length is measured by years estimated using a dated genealogy based on a relaxed molecular clocked estimated using Bayesian methods. (The results for alternative branch lengths are given in Tables 4–5, Figures 8–9 and Text S2.) We thus use a statistical modelling approach to explain the number 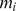 and size 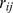 of HR events on a branch of length 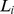 given the genealogy of a lineage.

### Description of models

We use a modelling approach to test whether recombination in *S. pneumoniae* is heterogeneous with regard to its rate or length distribution. Four models were devised to account for patterns observed in the data: (i) recombination is homogeneous in frequency and in size (Model 1); (ii) recombination is heterogeneous in frequency or in size, with heterogeneity modelled as deviation from the null model 1 (Model 2); (iii) recombination is heterogeneous in frequency and size, and is modelled by two independent and homogeneous processes of recombination with different frequency and size: micro-recombination and macro-recombination (Model 3); and (iv) recombination is heterogeneous in frequency and size, as in model 3, but the heterogeneity in frequency is independent from the heterogeneity in size (Model 4).

#### Model 1: A null model of recombination

**Figure 1:**
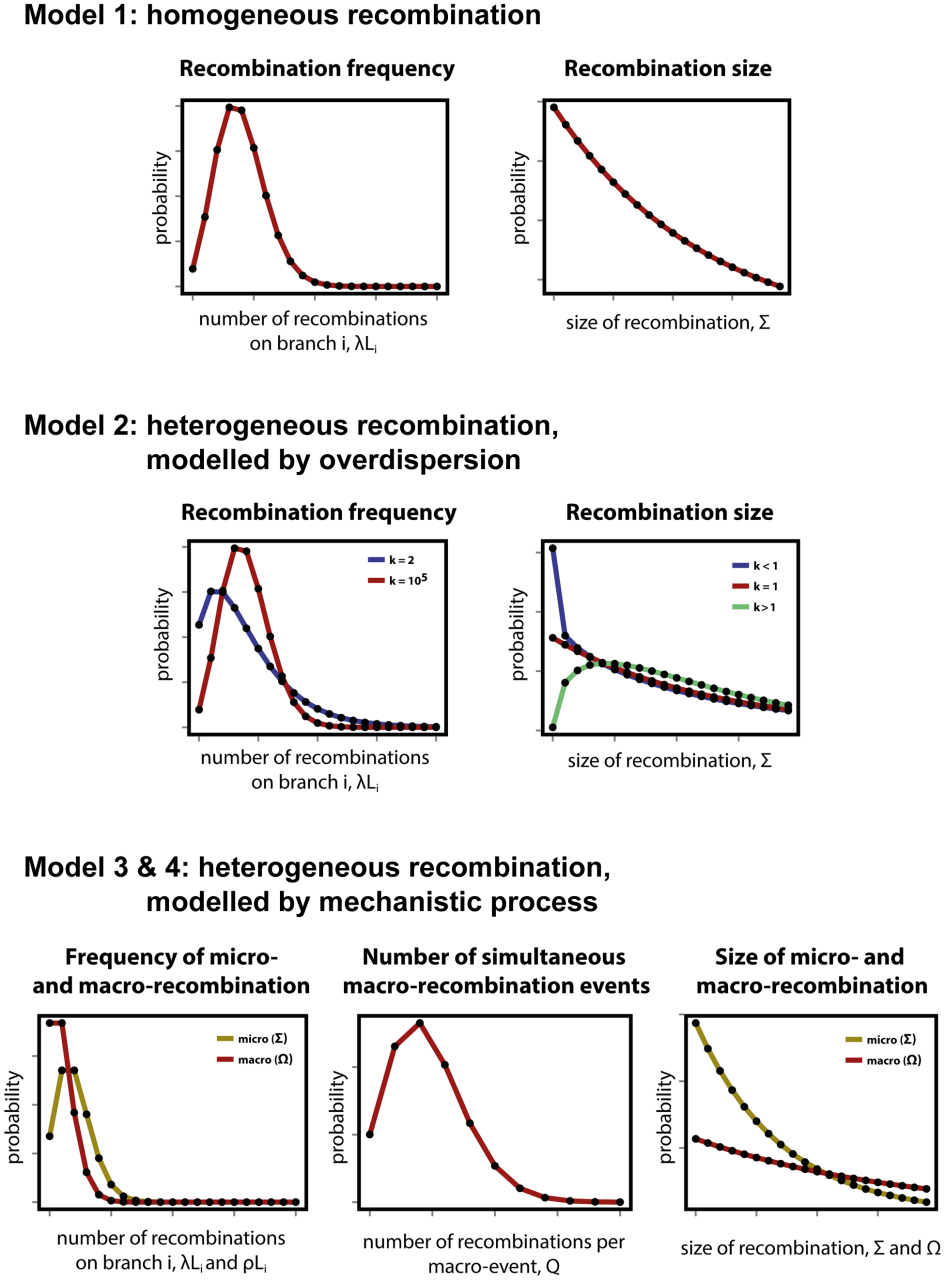
Modelling heterogeneity in pneumococcal data. (**Top row**) As a null expectation, recombination is modelled as a homogeneous process (Model 1). Frequency of recombination is determined by random encounters of DNA fragments by a bacteria cell, happening with a mean rate 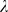. Recombinations arising through transformation terminate with a fixed per base probability, resulting in a geometric distribution of lengths with mean size 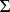. (**Middle row**) To account for heterogeneity in frequency and size, both the Poisson and geometric distributions are extended as negative binomial distributions with over-dispersion parameter 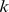 (Model 2). Heterogeneity in frequency will be reflected by a small value of 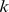 (blue), as large 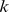 values return a Poisson distribution (red). Heterogeneity in size will be reflected by deviations from 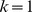, with over-representation of small fragments for 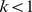 (blue) and over-representation of large fragments for 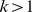 (red). (**Bottom row**) A formal model of heterogeneity of recombination distinguishes between micro-recombination (yellow; mean rate 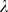 and size 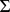) and macro-recombination (red; mean rate 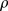, 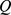 simultaneous events of mean size 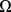). The difference between Model 3 and Model 4 is that the latter additionally assumes independency of frequency and size (in Model 4 macro-event has a probability 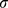 of being of mean size 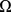).

The null expectation about the frequency and size distributions of homologous recombinations is that they are uniform. A priori, the transformation process can be envisaged by random encounters of DNA fragments by a bacterial cell. If one assumes such an encounter to be infrequent and independent of other encounters, then the transformation process is Poisson distributed with rate 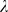. The size of the transformed fragments should follow a geometric distribution if they are equally likely to be fragmented at any given position during uptake of DNA by a competent cell. Indeed, *in vitro* homologous recombinations have been shown to fit a geometric distribution [13], parameterised by the mean recombination length parameter 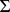, and such distributions are often priors for models which estimate recombination rates based on genetic data [21]. The shape of both probability distributions are displayed in Fig. 1, top row. This model implies that the mean number of recombinations on a branch of length 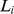 is going to be 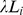, whereas the mean size of recombinations will be 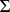.

#### Model 2: Heterogeneity of recombination, modelled by over-dispersion

The simplest way of modelling heterogeneity of a process is by quantifying a deviation from homogeneity by an over-dispersion. Over-dispersion, here denoted by parameter 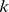, results from neglected unobserved variance in the studied phenomenon. Thus an extension of the homogeneous frequency model is a negative binomial distribution with mean 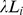 and over-dispersion parameter 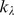, which becomes the Poisson distribution when 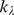 is infinitely large. The extension of the geometric size model is also a negative binomial distribution with over-dispersion parameter 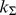, which becomes the geometric distribution when 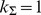. Both distributions are shown for different values of 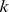 in Fig. 1, middle row, where the homogeneous distributions of model 1 are also shown by comparison.

#### Model 3: Heterogeneity of recombination, modelled by mechanistic process of micro- and macro-recombination

To be more explicit about heterogeneity of recombination we introduce a formal model with two classes of recombination: micro-recombination and macro-recombination. In this model, micro-recombination occurs at a rate 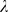 and results in a recombination event of mean size 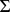. Macro-recombination occurs at rate 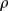, results in a mean 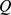 simultaneous recombination events of mean size 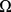. We assume both micro- and macro-recombination frequencies to be Poisson distributed, and the recombination lengths to be geometrically distributed. As the number of simultaneous macro-events 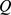 is Poisson distributed as well, the overall recombination frequency distribution is a convolution of three Poisson processes: one for micro-recombination and two for macro-recombination. The hypothetical distributions for micro- and macro-recombination are shown in Fig. 1, bottom row. (See supplementary Text S1 for the full derivation of the model.)

#### Model 4: Heterogeneity of recombination, modelled micro- and macro-recombination with no link between frequency and size

In the mixture model 3 of micro- and macro-recombination above both the rate of events and the size of events arise as mixtures, and these are linked by virtue of the fact that the macro-recombination process is assumed to give rise to both a larger number and bigger recombination events. To test whether the link between frequency and size is supported by the data, we consider a model which is identical in every way to model 3 except for the absence of this link. To this end, we introduce one extra parameter, denoted 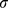, the probability that any given recombination event is large (with mean size 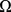) and not small (with mean size 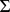).

### Model fitting

The models were fitted by the maximum likelihood method, namely maximising the log-likelihood function given in Text S1. This was done using optimization functions NMaximize or FindMaxiumum in Mathematica 8.0. The comparison between four different models was performed using the Akaike's Information Criterion, adjusted for finite degrees of freedom (AICc). We considered one model to be a better fit than another when the difference in AICc was less than 10 (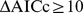). The best model was chosen as the one with the lowest value of 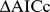. If multiple models were the best fit to the data, the model with the smallest number of parameters was chosen as the best by the rule of maximum parsimony. Goodness of fit was determined by verifying the ability of the model to replicate the data under re-simulation. To that end, marginal distributions of frequency and size of the simulations were compared to the equivalent marginal distributions of the data (see Results).

### Simulations

The details of the simulations are described in Text S3. In brief, an ancestral sequence of *S. pneumoniae* was chosen as the earliest isolate of PMEN1 known [19], [22]. A forward, discrete-time simulation was designed to simulate the evolution of the lineage, including diversification through recombination simulated through incorporating homologous sequence from other publically available pneumococcal genomes. We assumed that at every time step the sequence acquired a single base substitution, and could diversify into two independently diversifying lineages with a constant probability 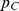. Each sequence also had a probability 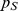 of being sampled at each timestep, after which it stopped evolving. The simulation was stopped when the population reached a maximal number of sequences, 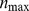. At each timestep, recombination occurred as prespecified by one of the four models: A, B, C or D. In Model A, recombination occurred homogeneously across the genome, with lengths of recombinations following a geometric distribution. In Model B, heterogeneity (micro/macro-recombination) was introduced in frequency but not the size. In Model C, heterogeneities in both frequency and size were correlated, as described in Model 3 above. In Model D, heterogeneity was also introduced in both frequency and size but the two were treated as independent variables for each recombination. Each model was run three times, giving 12 simulations overall.

## Results

### Heterogeneity of the recombination process

**Figure 2:**
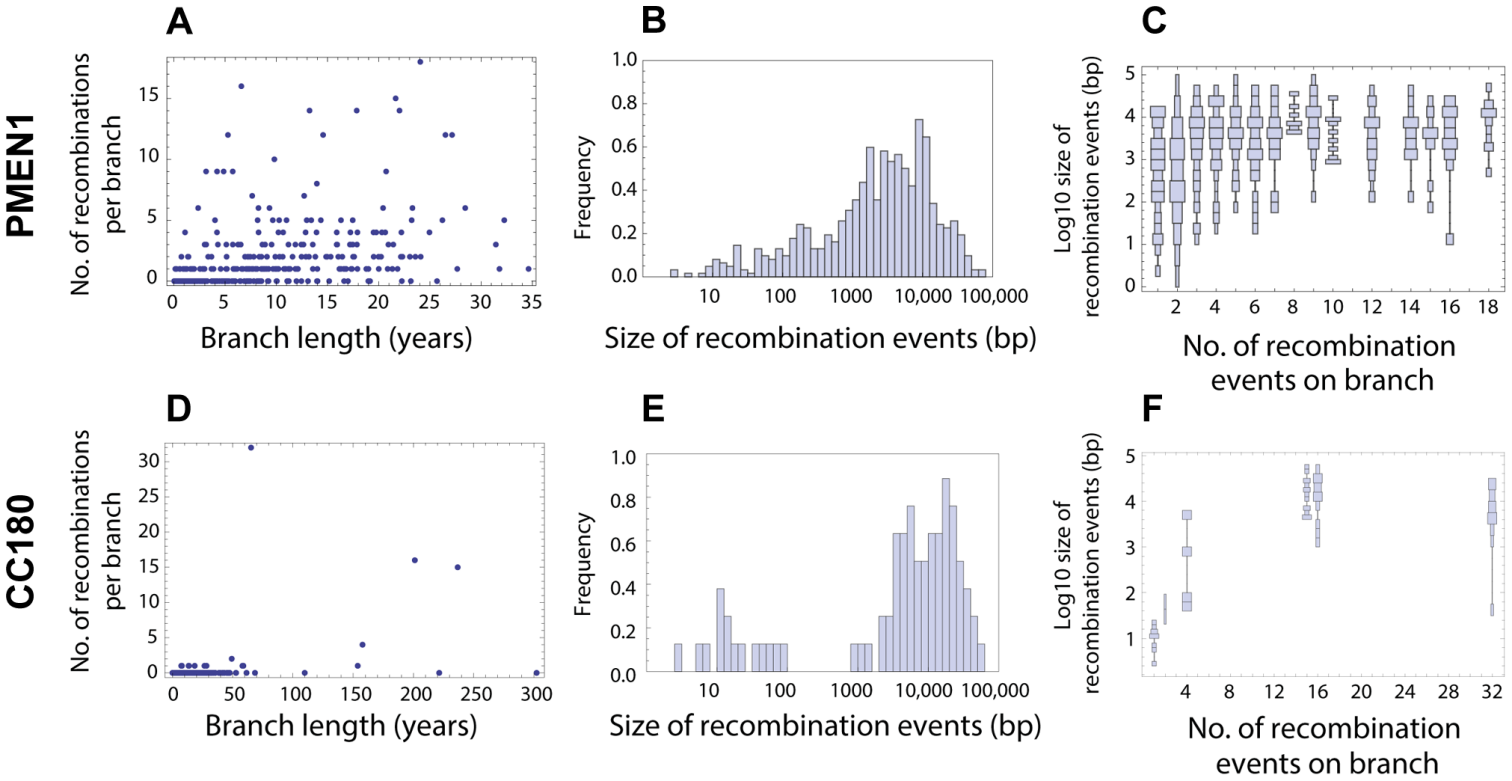
Distribution of recombination events as inferred from PMEN1 and CC180 genealogies. (A–C) Distribution of recombinations in PMEN1. (D–F) Distribution of recombinations in CC180. (**Left column**) Number of recombination events as a function of the branch length (years). The lengths were estimated by fitting a coalescent model of evolution to the heterochronously sampled sequences using BEAST. (**Middle column**) Frequency histogram of the size of the inferred recombination events. (**Right column**) Distribution chart of recombination event sizes: each vertical chart shows a distribution (histogram seen from above) of the recombination sizes for a given number of recombination events per tree branch. The goodness of fit of the four recombination models considered here is shown in Figures 3 and 4.

To study the process of HR in the evolutionary history of the two lineages, PMEN1 and CC180, we fitted mathematical models which describe how recombination events are distributed along the branches of the evolutionary tree of each lineage of *S. pneumoniae*. The procedure of model fitting is described in detail in Text S1. The phylogenies of both lineages have been constructed as described previously in [19], [20] based on vertically inherited point mutations, and were shown to be highly consistent with a molecular clock. Recombination events were reconstructed such that they were associated with particular branches of the phylogeny [19]. To remove events that may have been introduced through the movement of MGEs in PMEN1, rather than being mediated by HR, any events affecting the prophage remnant, prophage MM1-2008 or ICE *Sp*23FST81 were not considered in this analysis [22]. Likewise, for CC180, these MGEs included the 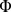
OXC141 prophage locus and a single putative integrative and conjugative element (ICE) [20]. The distribution of recombination events on the phylogenetic trees of both lineages is summarised in Fig. 2.

**Figure 3:**
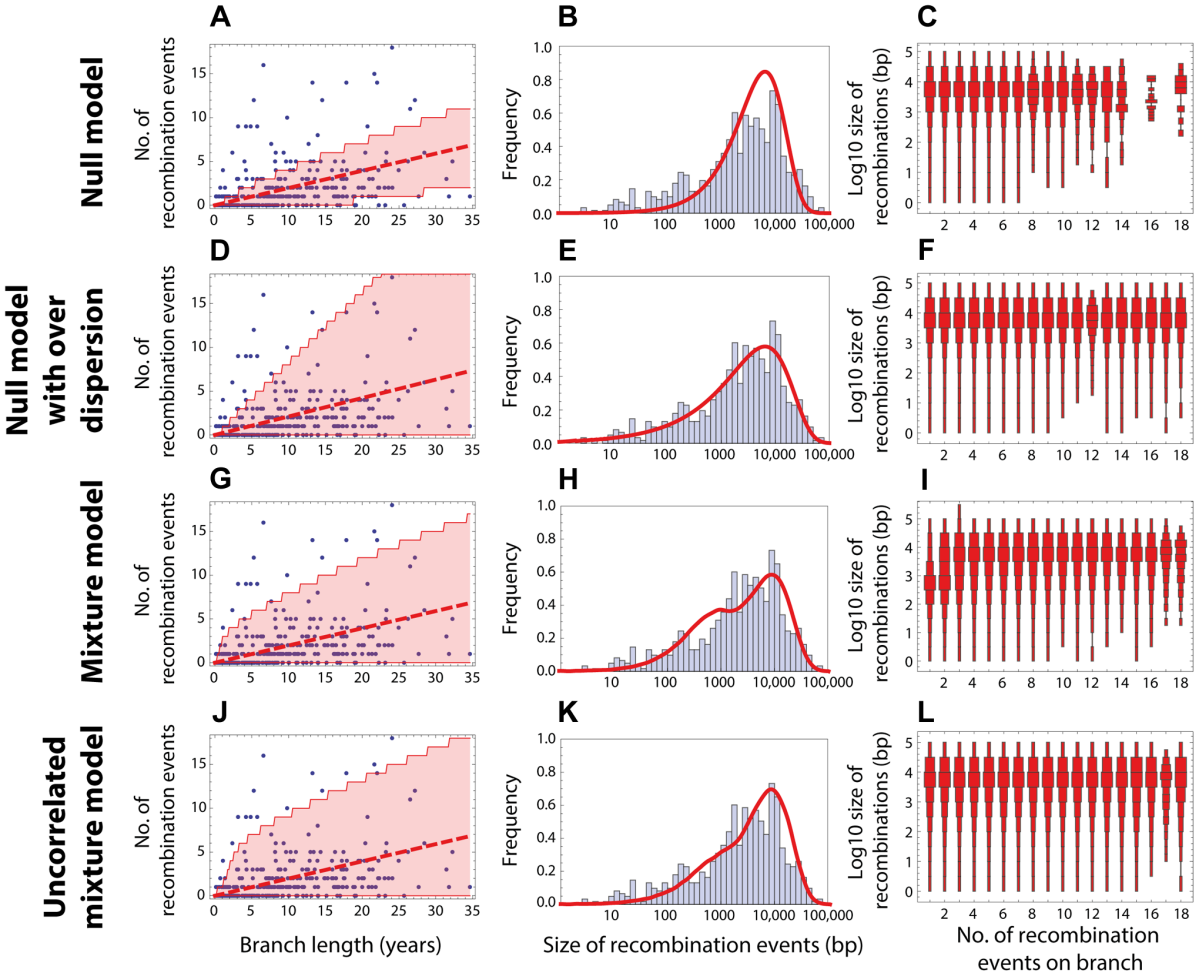
Goodness of fit for PMEN1 data. (Left column) Red dashed line shows the expected number of recombinations 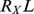, where 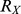 is the inferred maximum likelihood recombination rate for model 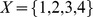, and 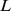 is the branch length; the red shaded areas show the 95% confidence interval. (Middle column) Red solid line shows the recombination size distribution as predicted by the maximum-likelihood model. (Right column) The size distributions of recombination events for a fixed number of events per tree branch (cf. Fig. 2C). This plot represents 100 replicates of the simulated distribution of recombination events given the observed PMEN1 phylogeny and the assumed model with best fit parameters. (A–C) Null model with homogeneous recombination (model 1, NM); 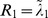. (D–F) Extended null model with over-dispersion (model 2, NMOD); 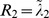. (G–I) Mixture model with micro- and macro-recombination (model 3, MM); 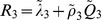. (J–L) Mixture model with no link between frequency and size of recombination events (model 4, UMM); 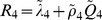.

**Figure 4:**
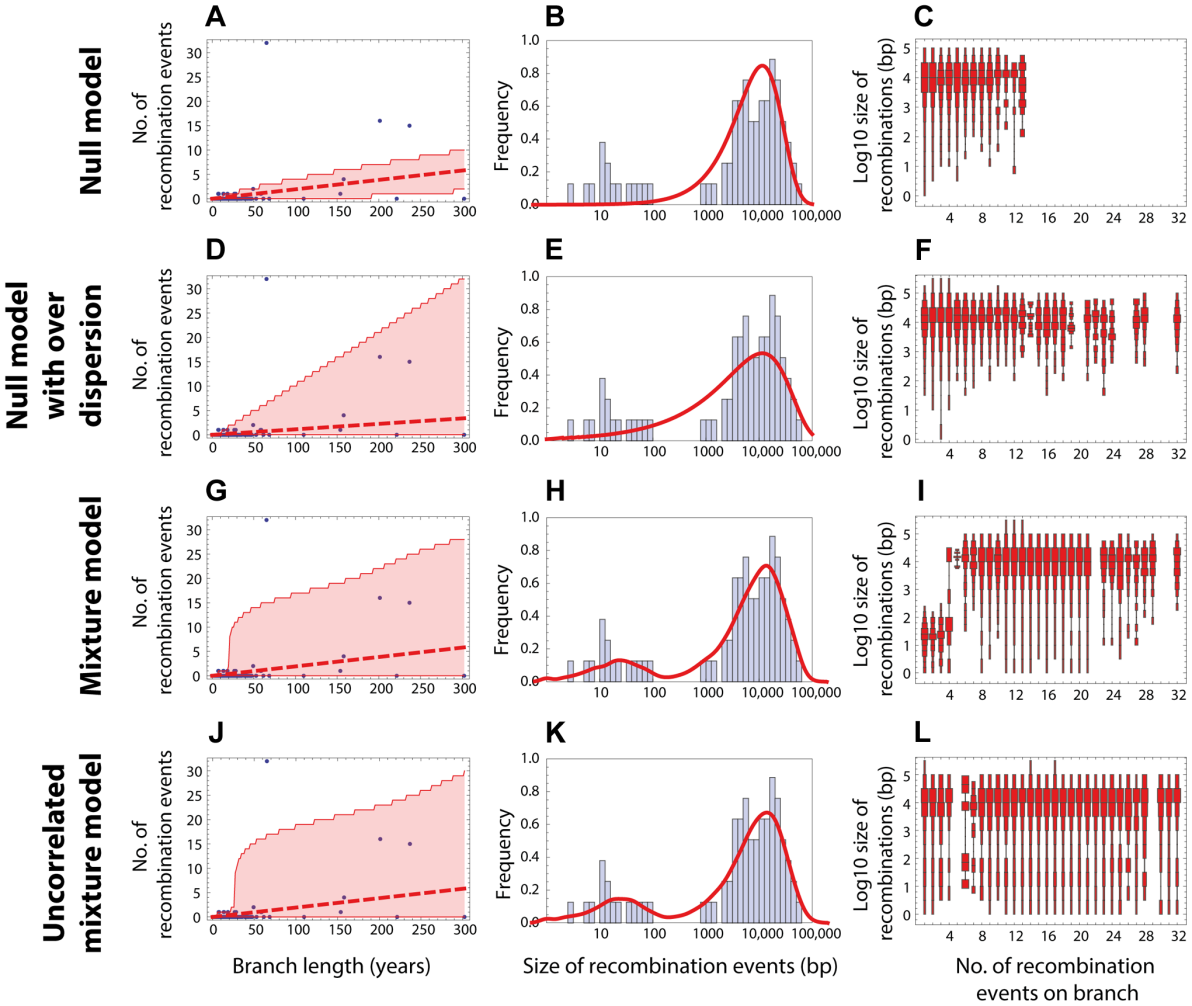
Goodness of fit for CC180 data. Blue: data, red: model. (A–C) Null model with homogeneous recombination (model 1, NM); (D–F) Extended null model with over-dispersion (model 2, NMOD); (G–I) Mixture model with micro- and macro-recombination (model 3, MM); (J–L) Mixture model with no link between frequency and size of recombination events (model 4, UMM). Data are displayed as in Fig. 3.

The simplest model considered is that recombination events occur as a homogeneous point Poisson process through time with rate 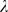, so that the number of events occurring on a genealogical branch of length 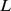 is Poisson distributed with mean 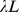, and that event sizes are geometrically distributed, with the mean length of genetic tract replaced by recombination for each event being 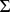 base pairs of DNA (see Fig. 1 and Methods). This model failed to capture clear heterogeneities in both the rate and size of events in PMEN1 (Fig. 3A–C & Table 1), and the same was true for the CC180 lineage (Fig. 4A–C & Table 2).

**Table 1:** Model comparison for PMEN1 data fit.

Model	AIC_c_	ΔAIC_c_	*n*	λ	Σ	*k_λ_*	*k*_Σ_	*ρ*	Ω	*Q*	*σ*
1 (NM)	13,488	528	2	0.20	6,400	–	–	–	–	–	–
2 (NMOD)	13,000	40	4	0.21	6,400	0.92	0.53	–	–	–	–
3 (MM)	12,960	0	5	0.058	580	–	–	0.060	8,800	2.3	–
4 (UMM)	13,001	40	6	0.11	160	–	–	0.016	7,700	5.1	0.83

The table summarises the goodness of fit of four models used to the distribution of recombinations on the PMEN1 phylogeny. The maximum likelihood estimates of the parameters are given for each of the four models (see Methods and Text S1). The goodness of fit of these models is compared by the use of Akaike's Information Criterion, adjusted for finite degrees of freedom (AICc). The number of parameters in the model is denoted 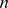, and the number of degrees of freedom in the data is 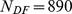. The mixture model (Model 3) provides the best fit (lowest AICc) of the recombination process in the genealogical reconstruction of the PMEN1 lineage. NM = Null model (model 1); NMOD = Null model with over-dispersion (model 2); MM = Mixture model (model 3); UMM = Uncorrelated mixture model (model 4).

**Table 2:** Model comparison for CC180 data fit.

Model	AIC_c_	ΔAIC_c_	*n*	λ	Σ	*k_λ_*	*k*_Σ_	*ρ*	Ω	*Q*	*σ*
1 (NM)	1,988	340	2	0.019	11,000	–	–	–	–	–	–
2 (NMOD)	1,741	93	4	0.011	11,000	0.10	0.47	–	–	–	–
3 (MM)	1,648	0	5	0.0029	27	–	–	0.0013	14,000	13	–
4 (UMM)	1,703	56	6	0.0044	26	–	–	0.00097	14,000	15	0.82

The table summarises the goodness of fit of four models used to the distribution of recombinations on the CC180 phylogeny. The layout of the table is identical to the one used in Table 1. 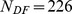.

A standard way to empirically describe heterogeneity is to quantify over-dispersion of the distribution of interest. To quantify heterogeneity in frequency and size in both lineages, we extended the approach in model 1. The extension of Poisson and geometric distribution is in both cases a negative binomial distribution with parameter 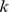, which reduces to a geometric distribution for 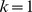 and to Poisson for very large values of 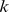 (see Fig. 1). A model based on a negative binomial distribution of events per branch with mean 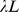 and dispersion coefficient 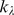, and a negative binomial distribution of event sizes with mean 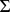 bp and dispersion coefficient 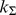 fit the data much better than the homogeneous, Poisson-based model for the PMEN1 dataset (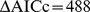; Fig. 3D–F & Table 1) and also for the CC180 dataset (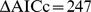; Fig. 4D–F & Table 2). This demonstrates that both the recombination rate and recombination event size are heterogeneous processes, but gives little insight into the potential mechanisms generating heterogeneity.

Heterogeneity in the recombination rate suggests that recombination sometimes occurs in discrete saltations rather than at a homogeneous rate. We further observed a correlation between the frequency of recombination events and their size (Fig. 2C and 2F). We thus modelled the recombination process by a mixture of two, homogeneous recombination processes. The first process, which we refer to as micro-recombination, leads to single small replacements. The second process, which we refer to as macro-recombination, leads to multiple synchronous or near-synchronous larger replacements. We assumed that the micro-recombination process is described by the same parameters 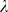 and 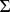 as in the null model; the macro-recombination process occurs at rate 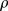, in which multiple tracts of DNA are incorporated into the genome by HR simultaneously (or at least in a short period of time compared to the genealogical branching process, so that these end up assigned to a single phylogenetic branch). We model the number of gene segments incorporated per macro-recombination event by a Poisson distribution with mean 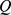, and the event sizes are geometrically distributed with mean length of genetic tract replaced by recombination for each event being 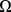 bp (see Fig. 1). In this model, the heterogeneity in rates is generated dynamically through the process of near-simultaneous recombination events, but this model alone does not generate excess heterogeneity in the size distribution of recombination event. The mixture model 3 provided a much better fit than the homogeneous model 1 for both PMEN1 lineage and CC180 lineage (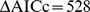 and 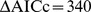, respectively). It also provided a better fit than the heterogeneous model 2 (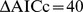 and 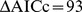), although results of comparing non-mechanistic descriptions of heterogeneity (Model 2) to mechanistic models (Model 3) should be interpreted with caution, since mechanistic models are likely to be more useful even for equivalent goodness of fit. (See also Figures 3G–I and 4G–I, Tables 1, 2 and 3.)

**Table 3:** Best fit parameters for the mixture model with micro- and macro-recombination (Model 3) with 95% confidence intervals.

Symbol	Description	ML estimate for PMEN1 (with 95% CI)	ML estimate for CC180 (with 95% CI)
*λ*	rate of MIC events [yr^−1^]	0.059 (0.044–0.072)	0.0029 (0.0016–0.0050)
Σ	mean size of MIC events [bp]	580 (350–840)	27 (15–52)
*ρ*	rate of MAC events [yr^−1^]	0.060 (0.045–0.080)	0.0013 (0.0004–0.0029)
Ω	mean size of MAC events [bp]	8,800 (7,800–10,100)	14,000 (11,000–19,000)
*Q*	mean number of MAC events	2.3 (1.8–2.9)	13 (9–16)

MIC = micro-recombination, MAC = macro-recombination, ML = maximum likelihood, CI = confidence interval.

A key property of the mixture model (Model 3) is that it generates correlation between the rate of recombination and the size of recombination events, since macro-recombination events, when they occur, are simultaneously larger and more numerous. To test whether this correlation was supported by the data, we compared the mixture model to a model identical in every respect, except for this correlation between rate and size (the uncorrelated mixture model 4). The resulting model fitted the data less well than the mixture model, with 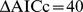 for PMEN1 data (Fig. 3J–L & Table 1) and 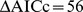 for CC180 data (Fig. 4J–L & Table 2).

**Figure 5:**
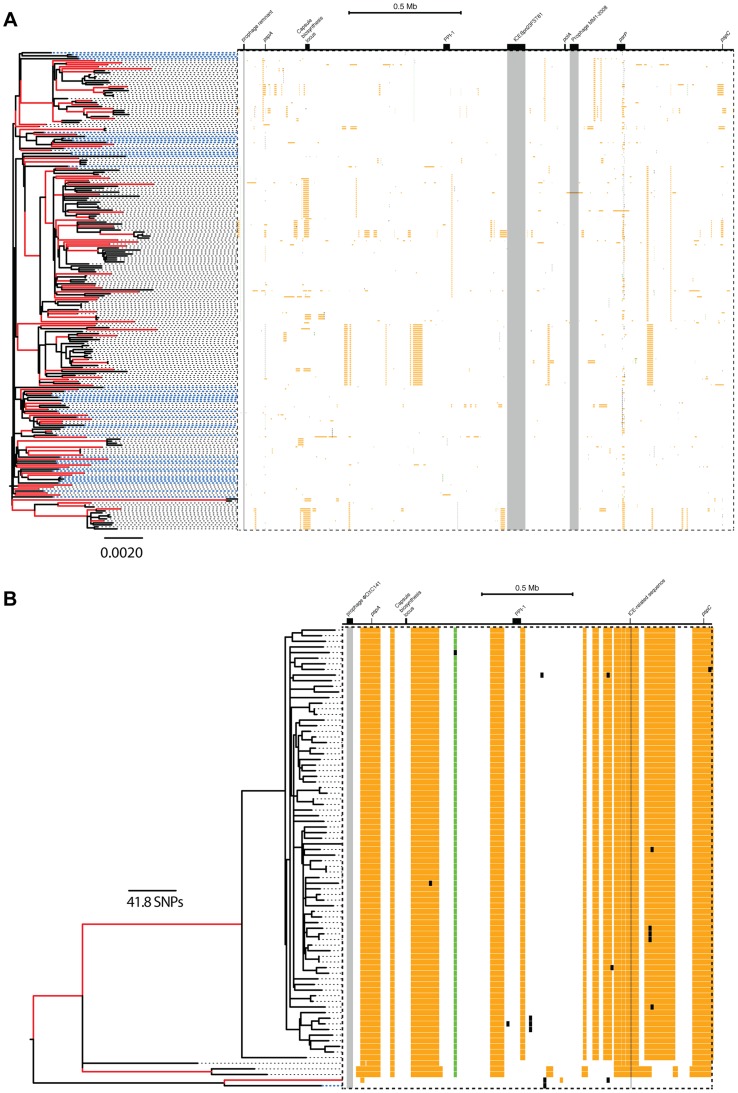
Distribution of micro- and macro-recombination events in the PMEN1 (A) and CC180 (B) phylogenies. In each panel, the layout is as follows. The maximum likelihood tree, constructed based on vertically inherited base substitutions, is displayed on the left. Recombinations were removed by identifying clusters of SNPs which cannot be explained by point mutations, as discussed in [19]. The branches at which only macro-recombinations are observed with the posterior probability of 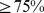 are coloured as red. The dashed blue lines correspond to isolates which have never undergone macro-recombination. On the right, the positions of recombination events per leaf of the phylogeny are displayed, with recombination events on internal branches appearing on multiple leaves. The panel shows the chromosomal locations of the putative recombination events detected in each terminal taxon. Yellow blocks denote recombinations inferred as macro-recombinations with the posterior probability of 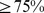. Black blocks denote recombinations inferred as micro-recombinations with the posterior probability of 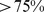. Green blocks denote all remaining recombinations.

**Figure 6:**
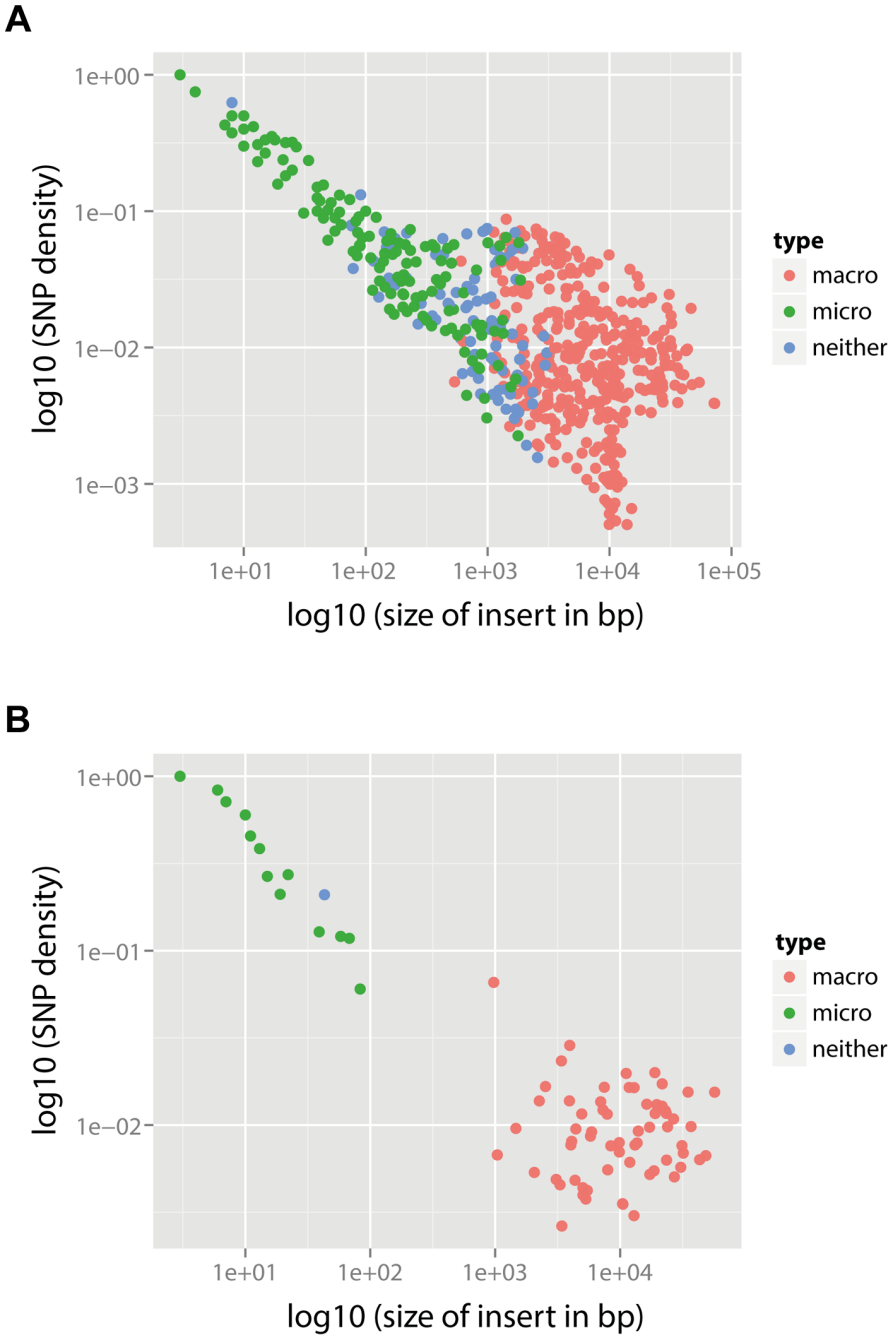
SNP density per branch versus the observed size of recombination events. (A) PMEN1 data. (B) CC180 data. Each point corresponds to a single recombination event (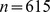). SNP density of each event is calculated as the number of SNPs within the event divided by the length of the event. The recombination events were distinguished according to their type based on the posterior probability 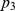 (see Text S1): macro-recombinations were defined as those with 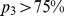 (red), micro-recombinations were defined as those with 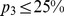 (green), and all the remaining ones were unclassified (blue).

In summary, the mechanistic mixture model 3 fit to the data well and generated novel mechanistic insight. These results were not dependent on the units used to measure branch length (see Methods and Text S2). Maximum likelihood estimates of the parameters and univariate 95% confidence intervals are given in Table 3. We then used this best fit model to determine the probability that each of the 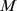 recombination events was generated either by micro-recombination or by macro-recombination. We found that of 615 events detected in PMEN1 lineage, 136 were 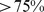 likely to have been generated by micro-recombination, and 389 were 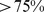 likely to have been generated by macro-recombination, with the remainder indeterminate. In CC180 lineage, of 79 events, 14 were 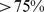 likely to have been generated by micro-recombination, and 64 were 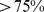 likely to have been generated by macro-recombination, with only one event indeterminate. The location of each event along the pneumococcal genome as well as in the inferred phylogeny of PMEN1 and CC180 lineage is shown in Figure 5. This figure shows the heterogeneity of recombination in the phylogenies of both lineages, where certain branches exhibit multiple, long macro-recombinations, whereas short, micro-recombinations tend to be more randomly distributed. This can also be seen in supplementary Figures 10 and 11 in Text S2, where an alternative distribution of recombination events in both lineages (i.e., all independent recombination events along the genome sorted by branch length) is shown. Finally, the distribution of micro- and macro-recombination events as a function of their length and the inferred number of SNPs is given in Figure 6. The figure shows that the inferred SNP density of micro- and macro-recombinations varies by approximately one order of magnitude, suggesting that the actual rate of micro-recombination may be considerably higher than that detectable through these data (but see Discussion).

In PMEN1, 10 serotype-switching events were observed [19] (i.e., those which induced a change from the serotype 23F to a different one), and all those events were found to be with 100% posterior probability likely to have been the result of macro-recombination. More generally, to examine whether recombinations at major antigen loci are likely macro-recombinations, we counted the number of recombinations spanning or overlapping five major antigen loci in PMEN1 (*pspA*, capsule biosynthesis locus, or *cps*, *pclA*, *psrP* and *pspC*) and three major antigen loci in CC180 (*pspA*, *cps*, and *pspC*). Of 171 such detected recombinations in PMEN1, 93 were 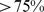 likely to have been generated by macro-recombination. By contrast, in CC180 only 4 recombinations at major antigens were found, however all 4 of them were 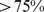 likely to have been generated by macro-recombination.

### Simulations of heterogeneity

To assess our method of detecting heterogeneity of recombination in the genetic data we designed a simulation framework where we evolved a pneumococcal lineage over time with four prespecified mechanisms of recombination, and examined how well we can distinguish between those mechanisms (see Methods and Text S3). Specifically, we designed analyses in which the PMEN1 reference genome diversified into a sample of related sequences through discrete time-steps as specified by one of four different simulation frameworks (Models A–D). We then reconstructed the evolutionary history of the lineage, with recombination events mapped onto the phylogeny, as described above and in [19]. We next fitted our four models of recombination (Fig. 1) to assess which of them best explains the underlying mechanism of diversification (see Tables 6–7 in Text S3). In the first simulation (A), recombination was simulated as a homogeneous process, and the homogeneous model 1 was the best fit. In the second simulation (B), the distinction between micro-recombination and macro-recombination was introduced but only based on frequency and not size, and in these cases model 3 was the best fit to the data. However, there was no significant difference in the size distributions between the two modes of recombination, contrasting with the fits to the genomic data. In the third simulation (C), a full mixture model of micro- and macro recombination was considered, and again model 3 was the best fit, with the likelihood of each model fits being of the same order of magnitude as in PMEN1 and CC180 data. Finally, in the fourth simulation (D), an uncorrelated mixture model was assumed with independent heterogeneity in frequency and size. In this case, in two runs there was no significant difference in the fit of model 3 and 4, while in the third model 4 was a much better fit to data than model 3. These simulations thus demonstrate that the observation of model 3 fitting the genomic data best, with a dramatic difference in lengths between the micro- and macro-recombinations, is unlikely to be an artefact of the method used to detect recombination, or the models' formulation

### Comparison with other empirical studies

**Figure 7:**
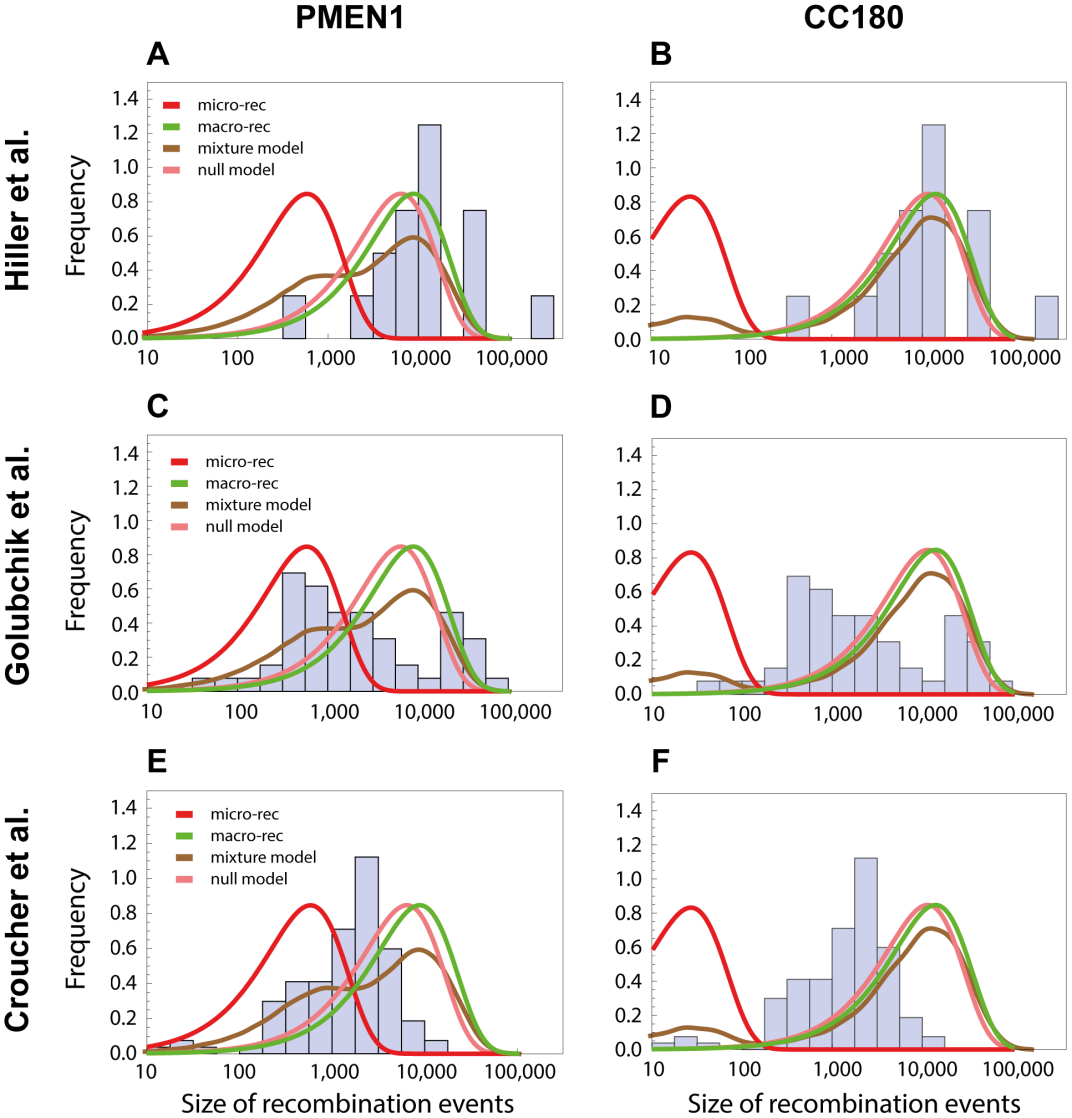
Comparison with recombinations detected by other methods. The length distribution of recombinations from other studies are compared with the length distributions of micro and macro recombinations inferred from PMEN1 (left column) or CC180 (right column). (A,B) The histogram shows a distribution of recombination sizes from an *in vivo* study, where 16 recombination events were collected from one patient suffering from pneumococcal infection over the period of 7 months [14]. Four lines correspond to four different functions based on the best-fit to the PMEN1 data (A) and CC180 data (B), as shown in Figs. 3 and 4, respectively: micro-recombination size model (red), macro-recombination size model (green), full size distribution of the mixture-model 3 (brown), and the homogeneous, null model (pink). (C,D) The histogram shows a distribution of recombination sizes from an epidemiological study where 53 recombination events of another lineage, ST695, were inferred [15]. The four lines are the same as above. (E,F) The histogram shows a distribution of recombination sizes from an *in vitro* transformation experiment [13]. The four lines are the same as above.

We next investigated whether the obtained results can explain recent observations of recombination in the pneumococcus using whole genome data. The near-simultaneous import of multiple fragments through transformation has previously been observed between a donor and recipient during a chronic infection *in vivo* in one patient [14], and also inferred through reconstructing the history of another lineage, sequence type 695 [15]. In the study by Hiller and colleagues [14], 16 recombination events varying in size from 0.4 kb to 235 kb (mean of 15 kb) were unidirectionally transferred from one donor strain into a recipient strain during an infection followed over a period of seven months. The observation that, in each case, multiple long recombinations had occurred over a defined short period suggested these examples might represent clear examples of the macro-recombination process. We found the size distribution of macro-recombinations to be in accordance with the one observed by Hiller et al. for both PMEN1 (see Fig. 7A) and CC180 lineage (see Fig. 7B).

On the other hand, the study by Golubchik et al. identified 53 recombination fragments in 5 vaccine escape recombinant lineages, ranging in size from 0.4 kb to 90 kb (mean of 10 kb). Although the distribution of recombination sizes inferred by this analysis of re-sequencing data did not resemble any of the distributions defined by the models of recombination presented here, it nevertheless suggests a strikingly heterogeneous recombination process (see Fig. 7C and 7D). A more formal approach would be needed to determine whether this is due to an actual recombination heterogeneity or due to another factor like the method used to infer recombination, or vaccine-induced selection (see also Discussion).

Finally, it has been demonstrated that multiple fragments of DNA can be imported by a member of the PMEN1 lineage during a single period of competence for transformation under controlled conditions [13]. While the overall distribution of sizes observed was similar to that reconstructed as happening during the lineage's diversification, there was less variation in the range of detected sizes. The discrepancy between the size distributions from the transformation experiment and the one observed in the PMEN1 lineage (see Fig. 7E) points to some interesting questions about varying conditions under which pneumococci undergo recombination during their evolution (see Discussion). Perhaps unsurprisingly, the predicted size distribution of the CC180 lineage was even less consistent with the distribution of recombinations from the *in vitro* experiment (see Fig. 7F).

### Saturation of the mismatch repair

One hypothesis that could explain the observed difference between micro- and macro-recombination could be the effect of mismatch repair (MMR; see also Discussion). MMR inhibits the acquisition of polymorphisms through transformation, but in the pneumococcus becomes saturated upon the import of around 150 SNPs [23], [24]. Thus micro-recombinations could be acquired under the constraint of this system, whereas macro-recombination could represent the acquisition of sequence unlimited by MMR. In accordance with this hypothesis, when we divided branches of the phylogeny on the basis of the most common mechanism of recombination occurring on them, those on which micro-recombination predominated generally imported fewer than 150 substitutions in total, while those on which macro-recombination was more common typically acquired many more than this (see Figures 12–13 and Text S2). We also examined whether there were differences in the types of substitutions introduced by micro- and macro-recombination, as MMR varies in the efficiency with which is repairs different mutations. We found that macro-recombinations were enriched for ‘low efficiency' markers, which are repaired most effectively by MMR both in PMEN1 (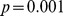), and in CC180 (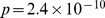). Interestingly, no association between the type of marker and the type of recombination was observed in the simulated pneumococcal sequences with preassumed micro- and macro-recombination mechanism (see Table 8 and Text S2).

## Discussion

Our analysis shows that both analysed lineages of *Streptococcus pneumoniae*, the multi-drug resistant PMEN1 and the older but less diverse CC180, have likely evolved under two distinct homologous recombination processes. The first process, which we call micro-recombination, occurred at a homogeneous clock-like rate and gave rise to isolated small genetic replacements. The second process, which we call macro-recombination, was more erratic, giving rise to large, multiple synchronous (or near-synchronous) replacements. While in PMEN1 we found both micro- and macro-recombinations to have occurred at a similar rate (every 17 years), in the less rapidly diversifying CC180 lineage micro-recombination was more frequent than macro-recombination (once in 340 years vs. once in 770 years). Overall, recombination was much more heterogeneous in CC180. Furthermore, the difference in sizes between micro- and macro-recombination was found to be greater in CC180 (0.03 kb vs. 14 kb) than in PMEN1 (0.6 kb vs. 9 kb). Finally, the number of simultaneous recombinations imported during macro-event was smaller in PMEN1 than in CC180 (2.3 vs. 15). The best fit parameters, together with the 95% confidence intervals, are summarised in Table 3.

The principal caveat in this analysis is that it is dependent on the correct identification of both the genealogy and the recombinations in the original analysis of the PMEN1 and CC180 lineages [19], [20]. The main evidence given for the correct identification of the recombinations is that their removal from the set of base substitutions used to construct the phylogeny results an improved ability to detect evidence of a molecular clock at a rate similar to other bacteria that do not undergo frequent homologous recombination [19], [25], the length distribution of putative events is similar to that detected experimentally [13], and that recombination events that can be inferred from phenotypic data (e.g., serotype switches) are predicted at the correct locus on the expected branch of the tree [12], [19]. However, we note that there is an inherent bias in the method described by Croucher et al., shared with other methods that use SNP density to detect recombination (e.g., maximum Chi-square method, ClonalFrame [21]), in that it is prone to missing short recombination events that happen to bring in few SNPs into the genome. Nonetheless, such events have a relatively small effect on estimates of branch length, and therefore estimates of the molecular clock rate. However, such bias means that we have likely under-estimated the rate of micro-recombination. This is best illustrated by comparing SNP density to the observed size of the recombination (Figure 6). The observed negative correlation between SNP density and recombination size (Spearman's rank correlation: 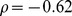, 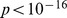 for PMEN1 and 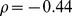, 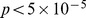 for CC180) is likely the result of the detection bias described above, and this suggests that we may lack the sensitivity to accurately quantify the rate of micro-recombination events. Simulations of the heterogeneity suggest that the actual rate of micro-recombination is likely to be roughly three times the estimated rate. Correspondingly, we found that the methods employed in this study were able to correctly identify the underlying model of evolution when simulations were performed under different models of diversification. This suggests that our observations are unlikely to be an artefact of the method used to detect recombination.

The presented analysis provides a quantitative model that could potentially explain other observations of recombination in the pneumococcus using whole genome data. The near-simultaneous import of multiple fragments through transformation has been observed previously in *in vivo* [14], [15] and *in vitro* studies [13]. We found that the micro/macro-recombination process could be consistent with size distributions of recombinations in some patient-derived sequences (cf. Fig. 7). However, there is weak evidence that this happens in the case of transformation *in vitro*. Therefore the observation of these two different types of recombination requires an explanation that can link the differences in properties and kinetics. It could be that genetic transformation through the competence system is only responsible for recombination through one of the modes, like micro-recombination, while other forms of bacterial “sex”, like conjugation or transduction, would lead to the acquisition of long stretches of DNA associated with macro-recombination. Conjugation has been observed to cause extensive sequence transfer in other streptococci, which would be consistent with this hypothesis conjugative transfer can result in multiple events if multiple conjugative origins are involved [26]. However, these exchanges are associated with *ori* sequences from conjugative elements, and therefore result in more regular recombination boundaries than are observed for the macro recombination events in this analysis [27]. Similarly, general transduction of sequence can import large DNA fragments of variable lengths, but typically only one can be packaged into a virion. As such mispackaging events are rare, this does not provide a likely explanation for the near-simultaneous import of multiple fragments [28].

Another potential explanation of the difference between micro- and macro-recombination may be how stretches of DNA are processed within the cell. For example, the recently identified competence-specific DNA-binding protein SsbB has been found capable of storing about 1.15 Mb of DNA imported by the competence system [27]. As the expression of this protein varies according to regulatory processes, it could play an important role in controlling the properties of recombination. However, given the comparatively homogeneous length distribution of recombinations observed in experimental transformation of the pneumococcus, it seems likely that extracellular degradation or intracellular processing are not the best candidates to explain the observed heterogeneity.

Hence it seems more likely that the observed dynamics represent transformation behaving in two distinct modes. One known threshold that could explain the variation is saturation of repair systems. MMR inhibits the acquisition of polymorphisms through transformation, but in the pneumococcus becomes saturated upon the import of around 150 SNPs [23], [24]. Here we found moderate but significant evidence for this hypothesis, which would suggest that it is the extent and type of DNA imported that triggers the switch between the two types of exchange. In the PMEN1 dataset, each homologous recombination imports a mean of 70 substitutions (116 substitutions for CC180), and *in vitro* experiments have demonstrated that multiple fragments can be imported simultaneously. Therefore the availability of high concentrations of divergent DNA, as observed in pneumococcal biofilms [29], or a state of ‘hyper-competence’, in which cells imported DNA more readily than normal, would seem likely to saturate the MMR system and potentially trigger the conditions required for macro-recombination.

The idea of the emergence of micro-recombination and macro-recombination via saturation of the MMR has the advantage that it is consistent with the observed positive correlation between frequency and size of recombinations (cf. Fig. 2C and 2F). Many macro-recombinations found in this study are considerably larger than any individual segment of donated sequence acquired by *S. pneumoniae in vitro*. This is likely to reflect the algorithm employed in the analysis of pneumococcal genomes, which clusters together nearby transformation events that originate from the same imported strand of DNA [13]. Therefore, integrating a larger number of imported sequence segments into the chromosome can both result in a greater number of distinct recombinations, and generate more extensive ‘mosaic’ events that would be reflected by an increase in the length of the overall transformation event in this analysis. Hence if a mechanism like MMR becomes saturated, it might not only result in more acquired recombinations but also in transformation of larger mosaic segments, resulting in a simple mechanistic link between frequency and size of recombinations. Interestingly, *in vitro* transformation experiments of pneumococcus, despite investigating transformation at two very different concentrations of exogenous DNA, did not find strong evidence for two distinct mechanisms of recombination [13]. This indicates that the observed difference may represent other environmental factors that affect the regulation of systems such as MMR.

It is also important to consider that the observed distribution of sequence is also the consequence of selection, which could be an alternative explanation for the observed heterogeneity. However, such a selection pressure would have to be highly generic to account for such a genome-wide phenomenon. One potential pressure that affects multiple loci, in particular several affected by a high density of recombinations, is immune-driven selection. Loci which are most likely to be under selective pressure of the immune system have been shown to be recombination hotspots [19]. As this selection is likely to be diversifying, it is conceivable that longer recombinations at these loci, inducing greater phenotypic changes, are under positive selection, and are thus more frequently observed. However, the mixture model 3 remains the best fit even after those events have been removed from the dataset (see Table 9–10 and Text S2). Therefore, we conclude that, even though immune selection is likely to play a role in shaping the distribution of recombination events in the pneumococcal genome, it is unlikely to explain the observed heterogeneity of homologous recombination in *S. pneumoniae*.

Another process that may skew the pattern of observed recombinations is the non-systematic nature of the isolate collections used in the original analyses. Two analyses were performed to assess the potential for biased sampling to affect the conclusions: the first excluded all isolates from the extensively sampled South African collection, while the second excluded all isolates serotyped as 19A to rule out potential vaccine induced selective pressure. In both cases, the results were qualitatively the same (Table 11 and Text S2).

In summary, we have firmly demonstrated that homologous recombination is heterogeneous, and found that the heterogeneity shows evidence of two modes of action, which we term micro- and macro-recombination. We have also found that saturation of the mismatch repair system is the most likely mechanism for inducing macro-recombination.

From a whole population survey, it has been observed that total homologous recombination rates vary substantially between pneumococcal lineages [12], and that an increased propensity for recombination is associated with increased antibiotic resistance [11]. Given this observation, it is particularly interesting that the two lineages studied here, that are at the opposite extremes in terms of their phenotype and evolutionary history, are both characterised by a highly heterogeneous recombination process. Furthermore, the aggregate recombination distribution sizes appear quite relatively consistent across different pneumococcal genotypes [12]. This all suggests that the micro- and macro-recombination are likely to play a role across the entire pneumococcal species. Based on the results presented here, it seems that micro-recombination is the more frequent process, whereas macro-recombination is likely to be the main driver of the bacterium's diversification.

How generally applicable these models are to the evolution of other species, and their relevance to wider questions about the evolution of homologous recombination itself [30], can be addressed as more genomic datasets become available.

## Supporting Information

Figure S1 Goodness of fit of the best-fitting mixture model 3 for two alternative branch length units (PMEN1). (A–C) Results for the underlying tree with branch lengths as substitution rates of the maximum likelihood estimate. (D–F) Results for the underlying tree with branch lengths as numbers of SNPs. Data are displayed as in Fig. 3G–H.
(TIF)

Figure S2 Goodness of fit of the best-fitting mixture model 3 for two alternative branch length units (CC180). (A–C) Results for the underlying tree with branch lengths as substitution rates of the maximum likelihood estimate. (D–F) Results for the underlying tree with branch lengths as numbers of SNPs. Data are displayed as in Fig. 3G–H.
(TIF)

Figure S3 Distribution of recombinations for PMEN1. Each horizontal line represents a full genome at each branch of the tree, and the blue squares correspond to the positions at which recombination events have been found. The lines are sorted according to the inferred branch length. Branches of the same length were plotted on a single line, with blue squares denoting positions at which recombinations have been detected at any of these branches. A single blue pixel corresponds to a window of the size 200 bp in which any recombination events have been detected.
(PNG)

Figure S4 Distribution of recombinations for CC180. Data are displayed as in Fig. 10.
(PNG)

Figure S5 Micro/macro-recombination vs. saturation of the mismatch repair (MMR) in PMEN1. Box-plots show the distribution of the number of SNPs in branches on which micro-recombinations occur (green) and those on which macro-recombinations occur (red). Two methods to classify branches were used: based on all events on a given branch being of the same type (left), or based on the predominating type on a given branch (right); branches failing to fulfil either condition were not plotted. The square diagram show the mean value per box-plot. The number of base substitutions previously identified as a MMR saturation threshold (150) is plotted as a black horizontal line.
(PDF)

Figure S6 Micro/macro-recombination vs. saturation of the mismatch repair (MMR) in CC180. Data are displayed as in Figure 12.
(PDF)

Table S1 Model comparison of four models for the PMEN1 tree with two alternative units of branch lengths. (A) Branch length is estimated using a substitution model in the maximum likelihood reconstruction of the genealogy. (B) Branch length is measured by the number of SNPs assigned to mutations along branch. Data are displayed as in Table 1.
(PDF)

Table S2 Model comparison of four models for the CC180 tree with two remaining units of branch lengths. (A) Branch length is estimated using a substitution model in the maximum likelihood reconstruction of the genealogy. (B) Branch length is measured by the number of SNPs assigned to mutations along branch. Data are displayed as in Table 1.
(PDF)

Table S3 Results of model fitting to simulated data. DNC = did not converge.
(PDF)

Table S4 Details of sequences used as sequence donors in simulations.
(PDF)

Table S5Heterogeneity of recombination versus ‘marker efficiency’. Markers were subdivided according to three types of substitutions considered: low-efficiency markers (transitions), mid-efficiency markers (transversions 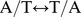), and high-efficiency markers (transversions 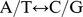 and 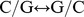). The lower the efficiency of a polymorphism, the higher the probability of being repaired by the MMR. In PMEN1 and CC180 we see a significant association between the two properties, namely macro-recombinations have more low-efficiency markers and less high-efficiency markers than expected from a random process. However, these associations are not observed in three simulations of micro- and macro-recombination.(PDF)

Table S6Model comparison of four models for recombinations occurring outside of five major antigen loci in PMEN1 (*pspA*, *cps*, *pclA*, *psrP* and *pspC*). Recombination events were removed when they fully spanned any of the loci, when they occurred within any of the loci or when they partially overlapped with any of the loci. The number of degrees of freedom in the data is 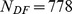. The layout of the table is identical to the one in Tables 1 and 2 in main text.(PDF)

Table S7Model comparison of four models for recombinations occurring outside of three major antigen loci in CC180 (*pspA*, *cps* and *pspC*) in analogy to Table 9. The number of degrees of freedom in the data is 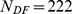.(PDF)

Table S8 Do isolate over-sampling or vaccine have any impact on the inference of heterogeneity? Two subdatasets were generated: (A) subset of data based on samples which did not come from Africa, and (B) subset of data based on samples which were not serotyped as 19A.
(PDF)

Text S1 Methods (full version).
(PDF)

Text S2 Additional results. Figures S1, S2, S3, S4, S5,S6 and Tables S1, S2 and S5, S6, S7, S8.
(PDF)

Text S3 Simulations of heterogeneity of recombination. Tables S3, S3 S4.
(PDF)
