# Supplementary figures and images for: Heterogeneity in the Frequency and Characteristics of Homologous Recombination in Pneumococcal Evolution
Source: PLoS Genet. 2014 May 1;10(5):e1004300. doi: 10.1371/journal.pgen.1004300 (PMC4006708; doi:10.1371/journal.pgen.1004300)

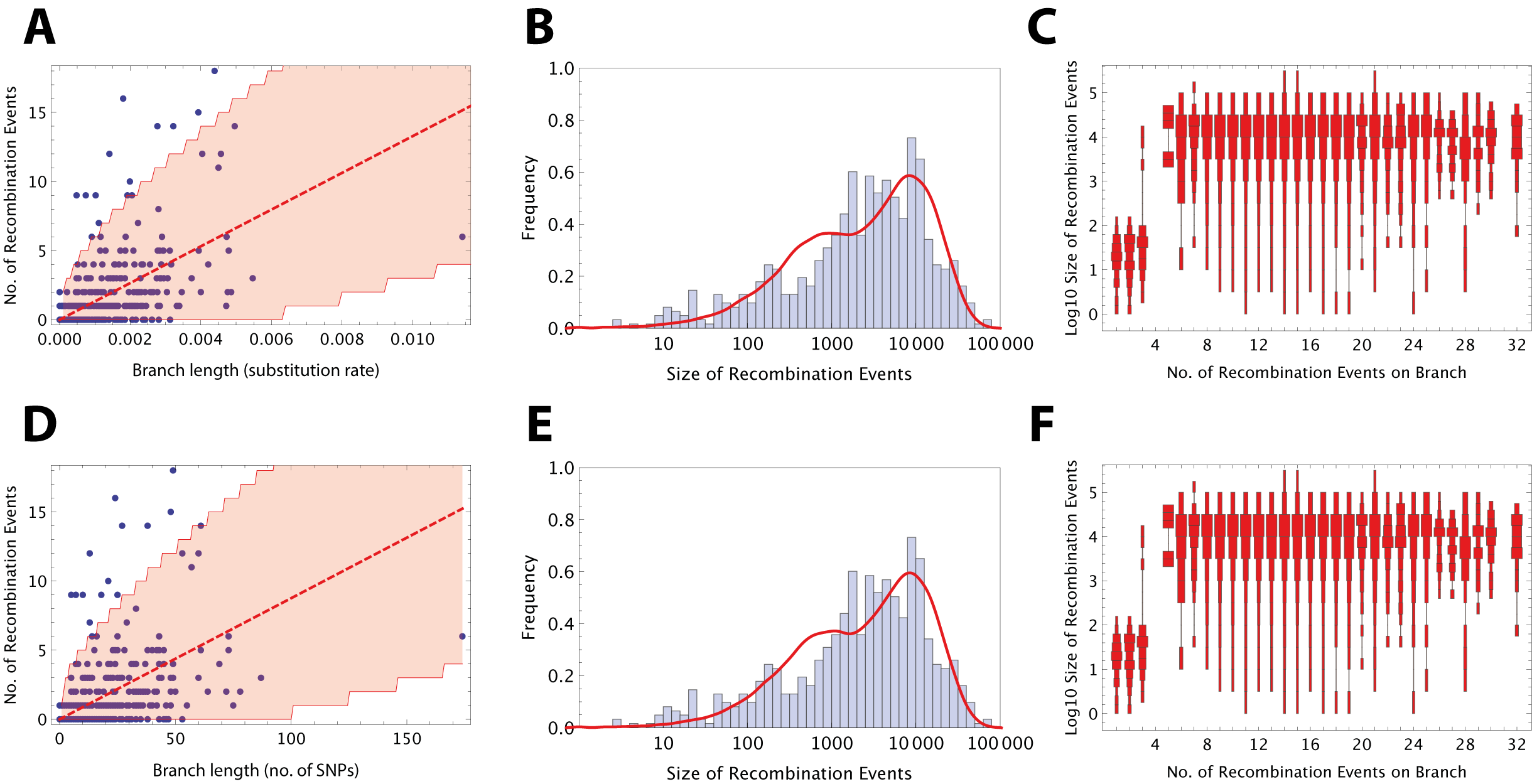

Supplement: Figure S1 — Goodness of fit of the best-fitting mixture model 3 for two alternative branch length units (PMEN1). (A–C) Results for the underlying tree with branch lengths as substitution rates of the maximum likelihood estimate. (D–F) Results for the underlying tree with branch lengths as numbers of SNPs. Data are displayed as in Fig. 3G–H. (TIF) [file pgen.1004300.s001.tif]

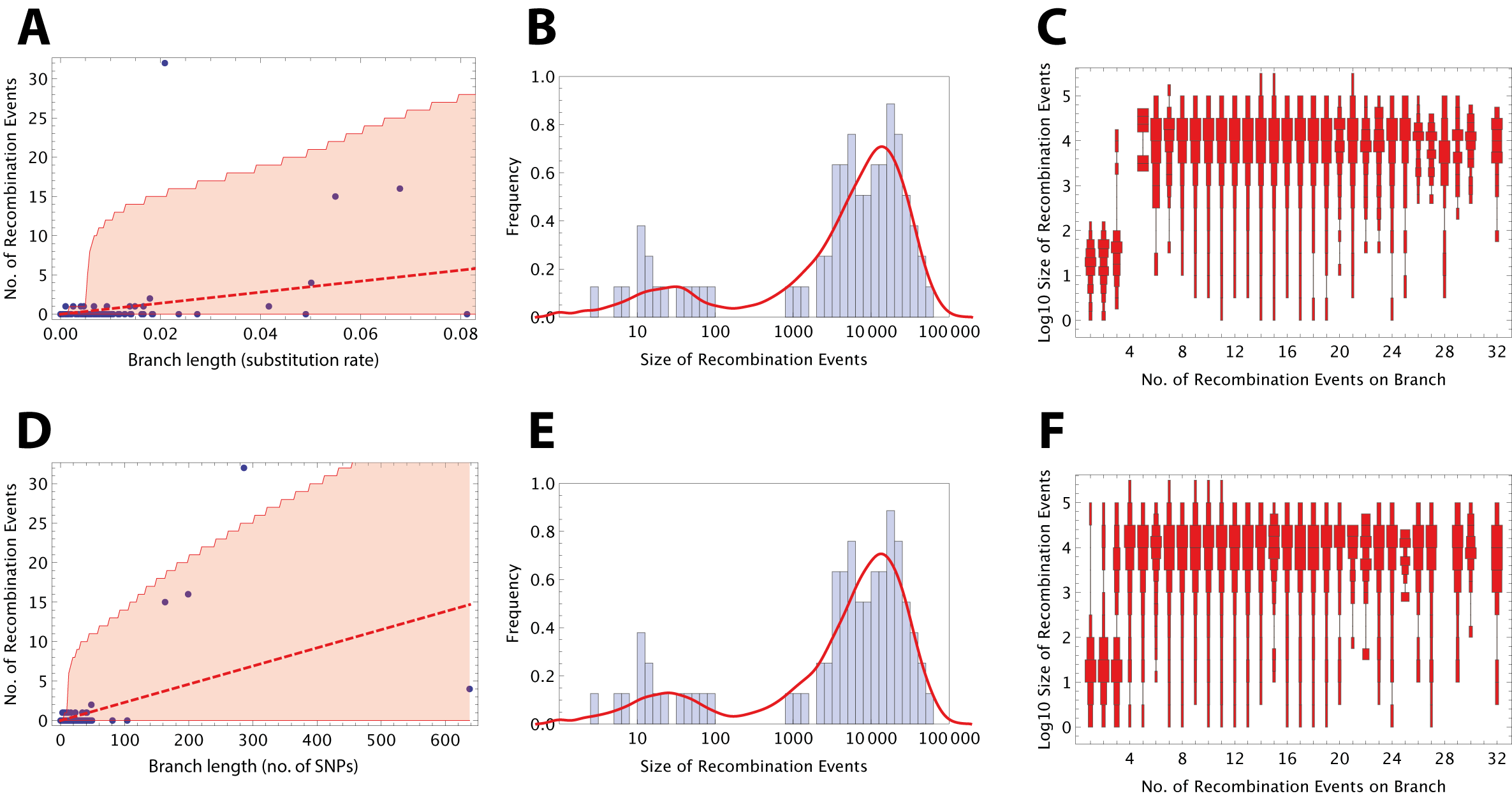

Supplement: Figure S2 — Goodness of fit of the best-fitting mixture model 3 for two alternative branch length units (CC180). (A–C) Results for the underlying tree with branch lengths as substitution rates of the maximum likelihood estimate. (D–F) Results for the underlying tree with branch lengths as numbers of SNPs. Data are displayed as in Fig. 3G–H. (TIF) [file pgen.1004300.s002.tif]

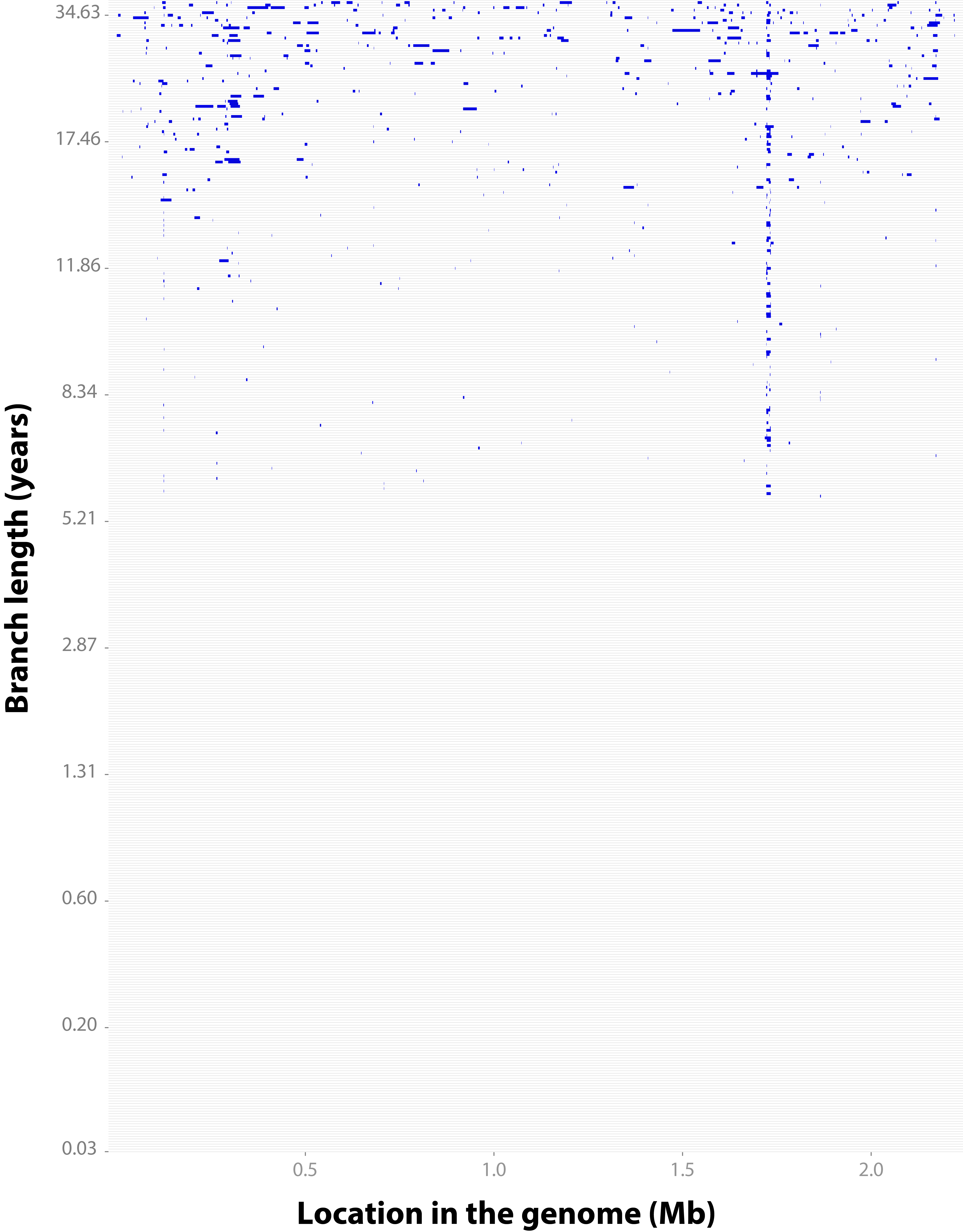

Supplement: Figure S3 — Distribution of recombinations for PMEN1. Each horizontal line represents a full genome at each branch of the tree, and the blue squares correspond to the positions at which recombination events have been found. The lines are sorted according to the inferred branch length. Branches of the same length were plotted on a single line, with blue squares denoting positions at which recombinations have been detected at any of these branches. A single blue pixel corresponds to a window of the size 200 bp in which any recombination events have been detected. (PNG) [file pgen.1004300.s003.png]

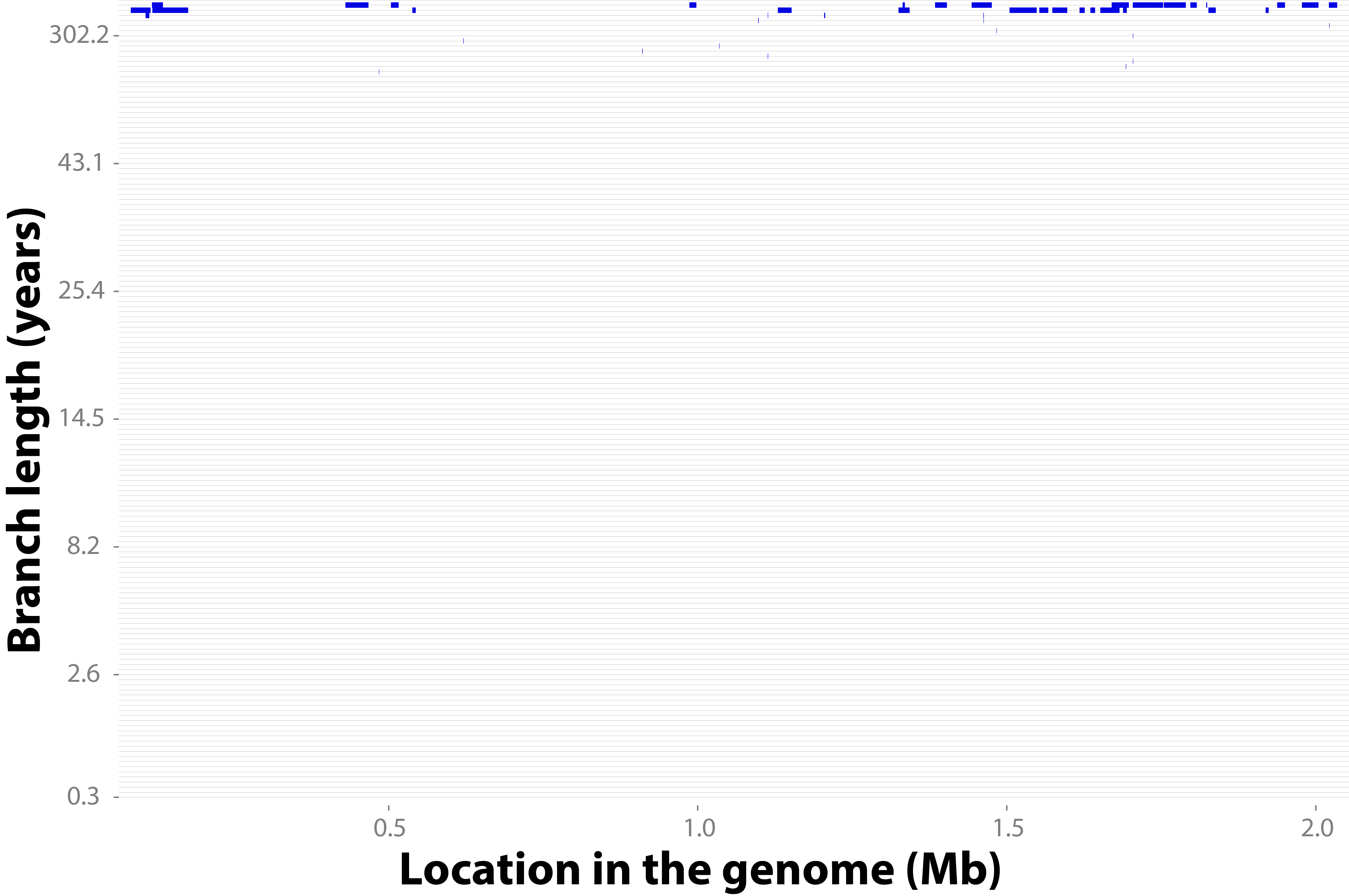

Supplement: Figure S4 — Distribution of recombinations for CC180. Data are displayed as in Fig. 10. (PNG) [file pgen.1004300.s004.png]

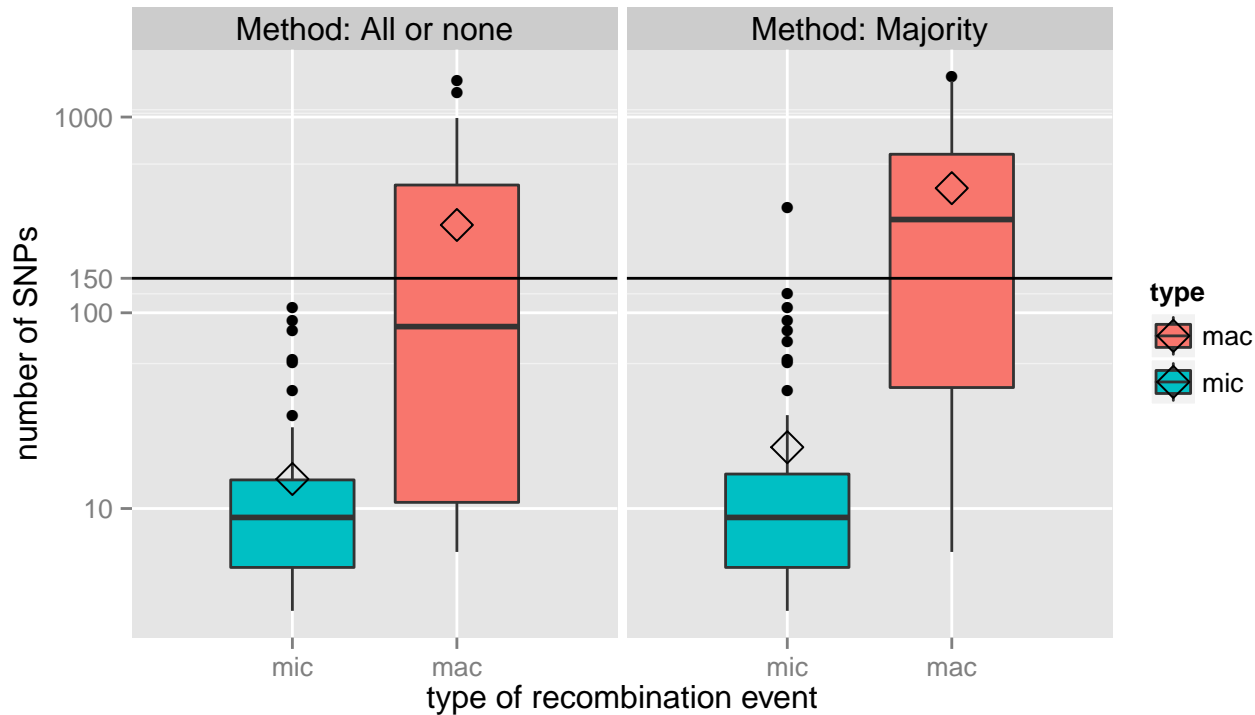

Supplement: Figure S5 — Micro/macro-recombination vs. saturation of the mismatch repair (MMR) in PMEN1. Box-plots show the distribution of the number of SNPs in branches on which micro-recombinations occur (green) and those on which macro-recombinations occur (red). Two methods to classify branches were used: based on all events on a given branch being of the same type (left), or based on the predominating type on a given branch (right); branches failing to fulfil either condition were not plotted. The square diagram show the mean value per box-plot. The number of base substitutions previously identified as a MMR saturation threshold (150) is plotted as a black horizontal line. (PDF) [file pgen.1004300.s005.pdf]

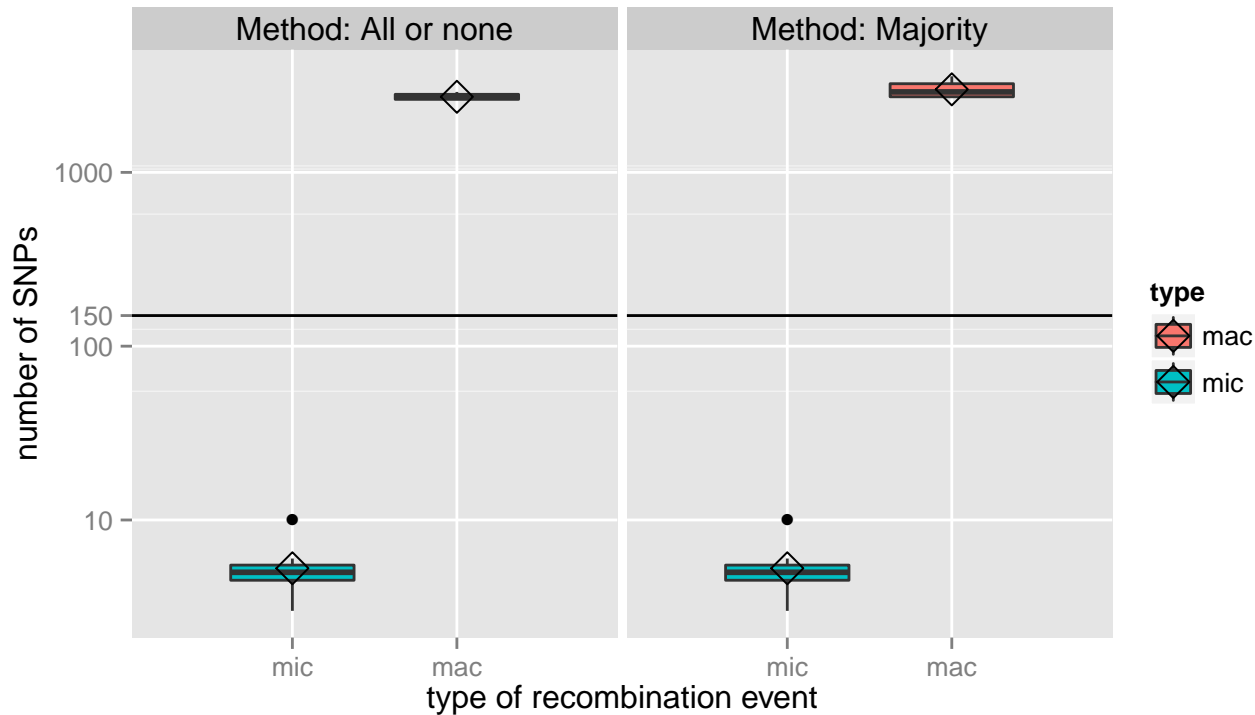

Supplement: Figure S6 — Micro/macro-recombination vs. saturation of the mismatch repair (MMR) in CC180. Data are displayed as in Figure 12. (PDF) [file pgen.1004300.s006.pdf]
