# Supplementary material for: Heterogeneity in the Frequency and Characteristics of Homologous Recombination in Pneumococcal Evolution
Source: PLoS Genet. 2014 May 1;10(5):e1004300. doi: 10.1371/journal.pgen.1004300 (PMC4006708; doi:10.1371/journal.pgen.1004300)
Supplement: Table S1 — Model comparison of four models for the PMEN1 tree with two alternative units of branch lengths. (A) Branch length is estimated using a substitution model in the maximum likelihood reconstruction of the genealogy. (B) Branch length is measured by the number of SNPs assigned to mutations along branch. Data are displayed as in Table 1. (PDF) [file pgen.1004300.s007.pdf]

A: MLE

**Model**	AIC_c	ΔAIC_c	λ	Σ	k_λ	k_Σ	ρ	Ω	Q	σ
1 (NM)	13,295	479	1300	6,400	–	–	–	–	–	–
2 (NMOD)	12,909	93	1300	6,400	1.4	0.53	–	–	–	–
3 (MM)	12,816	0	390	550	–	–	490	8,900	1.9	–
4 (UMM)	12,850	34	840	160	–	–	90	7,800	5.3	0.83

B: SNPs

**Model**	AIC_c	ΔAIC_c	λ	Σ	k_λ	k_Σ	ρ	Ω	Q	σ
1 (NM)	13,441	516	0.088	6,400	–	–	–	–	–	–
2 (NMOD)	12,966	41	0.095	6,400	0.99	0.53	–	–	–	–
3 (MM)	12,925	0	0.026	580	–	–	0.028	8,900	2.2	–
4 (UMM)	12,960	35	0.052	150	–	–	0.0066	7,700	5.4	0.83
