# Supplementary material for: Heterogeneity in the Frequency and Characteristics of Homologous Recombination in Pneumococcal Evolution
Source: PLoS Genet. 2014 May 1;10(5):e1004300. doi: 10.1371/journal.pgen.1004300 (PMC4006708; doi:10.1371/journal.pgen.1004300)
Supplement: Table S2 — Model comparison of four models for the CC180 tree with two remaining units of branch lengths. (A) Branch length is estimated using a substitution model in the maximum likelihood reconstruction of the genealogy. (B) Branch length is measured by the number of SNPs assigned to mutations along branch. Data are displayed as in Table 1. (PDF) [file pgen.1004300.s008.pdf]

**A: MLE**

| **Model** | $AIC_c$ | $\Delta AIC_c$ | $\lambda$ | $\Sigma$ | $k_\lambda$ | $k_\Sigma$ | $\rho$ | $\Omega$ | Q | $\sigma$ |
|---|---|---|---|---|---|---|---|---|---|---|
| 1 (NM) | 1,973 | 329 | 70 | 11,000 | – | – | – | – | – | – |
| 2 (NMOD) | 1,738 | 94 | 40 | 11,000 | 0.12 | 0.47 | – | – | – | – |
| 3 (MM) | 1,644 | 0 | 10 | 27 | – | – | 4.7 | 14,000 | 13 | – |
| 4 (UMM) | 1,699 | 55 | 17 | 26 | – | – | 3.5 | 14,000 | 15 | 0.82 |

**B: SNPs**

| **Model** | $AIC_c$ | $\Delta AIC_c$ | $\lambda$ | $\Sigma$ | $k_\lambda$ | $k_\Sigma$ | $\rho$ | $\Omega$ | Q | $\sigma$ |
|---|---|---|---|---|---|---|---|---|---|---|
| 1 (NM) | 1,878 | 232 | 0.023 | 11,000 | – | – | – | – | – | – |
| 2 (NMOD) | 1,731 | 84 | 0.011 | 11,000 | 0.22 | 0.47 | – | – | – | – |
| 3 (MM) | 1,646 | 0 | 0.0034 | 27 | – | – | 0.0024 | 14,000 | 8 | – |
| 4 (UMM) | 1,690 | 44 | 0.0057 | 26 | – | – | 0.0012 | 14,000 | 15 | 0.82 |
