# Supplementary material for: Heterogeneity in the Frequency and Characteristics of Homologous Recombination in Pneumococcal Evolution
Source: PLoS Genet. 2014 May 1;10(5):e1004300. doi: 10.1371/journal.pgen.1004300 (PMC4006708; doi:10.1371/journal.pgen.1004300)
Supplement: Table S3 — Results of model fitting to simulated data. DNC = did not converge. (PDF) [file pgen.1004300.s009.pdf]

**Model**	**Run**	**ΔAIC$_c$ Model1**	**ΔAIC$_c$ Model 2**	**ΔAIC$_c$ Model 3**	**ΔAIC$_c$ Model 4**	**Mean micro**	**Mean macro**
A	1	**0**	3	6	9	5,200	9,200
A	2	**0**	1	DNC	8	NA	NA
A	3	**0**	2	DNC	9	NA	NA
B	1	119	28	**0**	1	5,900	5,900
B	2	120	21	**0**	2	5,500	5,500
B	3	125	26	**0**	4	4,900	6,800
C	1	256	61	**0**	34	570	8,000
C	2	250	46	**0**	43	960	8,400
C	3	184	46	**0**	19	680	9,400
D	1	69	11	**6**	0	400	8,000
D	2	115	42	**0**	9	620	9,100
D	3	78	33	39	**0**	6,100	6,100
