# Supplementary material for: Heterogeneity in the Frequency and Characteristics of Homologous Recombination in Pneumococcal Evolution
Source: PLoS Genet. 2014 May 1;10(5):e1004300. doi: 10.1371/journal.pgen.1004300 (PMC4006708; doi:10.1371/journal.pgen.1004300)
Supplement: Table S4 — Details of sequences used as sequence donors in simulations. (PDF) [file pgen.1004300.s010.pdf]

**Genome**	**Accession code**
Streptococcus pneumoniae 670-6B	CP002176
Streptococcus pneumoniae 70585	CP000918
Streptococcus pneumoniae AP200	CP002121
Streptococcus pneumoniae CGSP14	CP001033
Streptococcus pneumoniae D39	CP000410
Streptococcus pneumoniae G54	CP001015
Streptococcus pneumoniae gamPNI0373	CP001845
Streptococcus pneumoniae Hungary19A-6	CP000936
Streptococcus pneumoniae INV104	FQ312030
Streptococcus pneumoniae INV200	FQ312029
Streptococcus pneumoniae JJA	CP000919
Streptococcus pneumoniae OXC141	FQ312027
Streptococcus pneumoniae P1031	CP000920
Streptococcus pneumoniae R6	AE007317
Streptococcus pneumoniae ST556	CP003357
Streptococcus pneumoniae Taiwan19F-14	CP000921
Streptococcus pneumoniae TCH8431/19A	CP001993
Streptococcus pneumoniae TIGR4	AE005672
