# Supplementary material for: Heterogeneity in the Frequency and Characteristics of Homologous Recombination in Pneumococcal Evolution
Source: PLoS Genet. 2014 May 1;10(5):e1004300. doi: 10.1371/journal.pgen.1004300 (PMC4006708; doi:10.1371/journal.pgen.1004300)
Supplement: Table S5 — Heterogeneity of recombination versus ‘marker efficiency’. Markers were subdivided according to three types of substitutions considered: low-efficiency markers (transitions), mid-efficiency markers (transversions ), and high-efficiency markers (transversions and ). The lower the efficiency of a polymorphism, the higher the probability of being repaired by the MMR. In PMEN1 and CC180 we see a significant association between the two properties, namely macro-recombinations have more low-efficiency markers and less high-efficiency markers than expected from a random process. However, these associations are not observed in three simulations of micro- and macro-recombination. (PDF) [file pgen.1004300.s011.pdf]

PMEN1 ($p = 0.001$**)**

		type of substitution		
		low	mid	high
**type of rec.**	micro	1,235	159	418
	macro	28,466	3,376	7,778

CC180 ($p = 2.36 \times 10^{-10}$**)**

		type of substitution		
		low	mid	high
**type of rec.**	micro	29	15	29
	macro	6,666	644	1,780

Simulation: run 1 ($p = 0.3$**)**

		type of substitution		
		low	mid	high
**type of rec.**	micro	137	9	37
	macro	18,391	2,034	4,865

Simulation: run 2 ($p = 0.39$**)**

		type of substitution		
		low	mid	high
**type of rec.**	micro	251	24	63
	macro	16,695	2,040	4,754

Simulation: run 3 ($p = 0.94$)

		type of substitution		
		low	mid	high
**type of rec.**	micro	194	23	52
	macro	15,407	1,932	4,301
