# Supplementary material for: Heterogeneity in the Frequency and Characteristics of Homologous Recombination in Pneumococcal Evolution
Source: PLoS Genet. 2014 May 1;10(5):e1004300. doi: 10.1371/journal.pgen.1004300 (PMC4006708; doi:10.1371/journal.pgen.1004300)
Supplement: Table S6 — Model comparison of four models for recombinations occurring outside of five major antigen loci in PMEN1 (pspA, cps, pclA, psrP and pspC). Recombination events were removed when they fully spanned any of the loci, when they occurred within any of the loci or when they partially overlapped with any of the loci. The number of degrees of freedom in the data is . The layout of the table is identical to the one in Tables 1 and 2 in main text. (PDF) [file pgen.1004300.s012.pdf]

**Model**	AIC_c	ΔAIC_c	λ	Σ	k_λ	k_Σ	ρ	Ω	Q	σ
1 (NM)	9,935	501	0.14	6,100	–	–	–	–	–	–
2 (NMOD)	9,493	68	0.15	6,100	0.47	0.59	–	–	–	–
3 (MM)	9,425	0	0.037	770	–	–	0.033	8,000	3.2	–
4 (UMM)	9,505	79	0.047	220	–	–	0.026	7,200	3.7	0.84
