# Supplementary material for: Heterogeneity in the Frequency and Characteristics of Homologous Recombination in Pneumococcal Evolution
Source: PLoS Genet. 2014 May 1;10(5):e1004300. doi: 10.1371/journal.pgen.1004300 (PMC4006708; doi:10.1371/journal.pgen.1004300)
Supplement: Table S7 — Model comparison of four models for recombinations occurring outside of three major antigen loci in CC180 (pspA, cps and pspC) in analogy to Table 9. The number of degrees of freedom in the data is . (PDF) [file pgen.1004300.s013.pdf]

**Model**	AIC_c	ΔAIC_c	λ	Σ	k_λ	k_Σ	ρ	Ω	Q	σ
1 (NM)	1,873	321	0.018	10,000	–	–	–	–	–	–
2 (NMOD)	1,643	92	0.011	10,000	0.11	0.46	–	–	–	–
3 (MM)	1,552	0	0.0030	27	–	–	0.0012	13,000	12	–
4 (UMM)	1,607	55	0.0044	26	–	–	0.00097	13,000	14	0.81
