# Supplementary material for: Heterogeneity in the Frequency and Characteristics of Homologous Recombination in Pneumococcal Evolution
Source: PLoS Genet. 2014 May 1;10(5):e1004300. doi: 10.1371/journal.pgen.1004300 (PMC4006708; doi:10.1371/journal.pgen.1004300)
Supplement: Table S8 — Do isolate over-sampling or vaccine have any impact on the inference of heterogeneity? Two subdatasets were generated: (A) subset of data based on samples which did not come from Africa, and (B) subset of data based on samples which were not serotyped as 19A. (PDF) [file pgen.1004300.s014.pdf]

A: African samples excluded

**Model**	AIC_c	ΔAIC_c	λ	Σ	k_λ	k_Σ	ρ	Ω	Q	σ
1 (NM)	10,820	435	0.18	6,600	–	–	–	–	–	–
2 (NMOD)	10,429	45	0.19	6,600	1.0	0.52	–	–	–	–
3 (MM)	10,384	0	0.061	680	–	–	0.050	9,400	2.4	–
4 (UMM)	10,437	53	0.11	270	–	–	0.012	8,200	6.0	0.78

B: 19A serotypes excluded

**Model**	AIC_c	ΔAIC_c	λ	Σ	k_λ	k_Σ	ρ	Ω	Q	σ
1 (NM)	12,472	491	0.19	6,300	–	–	–	–	–	–
2 (NMOD)	12,011	30	0.20	6,300	0.93	0.53	–	–	–	–
3 (MM)	11,980	0	0.059	600	–	–	0.058	8,800	2.3	–
4 (UMM)	12,010	30	0.12	150	–	–	0.012	7,700	6.1	0.82
