# Supplementary material for: Heterogeneity in the Frequency and Characteristics of Homologous Recombination in Pneumococcal Evolution
Source: PLoS Genet. 2014 May 1;10(5):e1004300. doi: 10.1371/journal.pgen.1004300 (PMC4006708; doi:10.1371/journal.pgen.1004300)
Supplement: Text S1 — Methods (full version). (PDF) [file pgen.1004300.s015.pdf]

Text S1. Methods (full version).

1 Structure of data

The analysis presented is based on the HR events previously described by Croucher et al. (2011). Briefly, that analysis identified M independent HR events as clusters of SNPs in a genealogy reconstructed from N whole genome alignments of the PMEN1 lineage. The reconstructed genealogy has B branches. Let $i = 1, ..., B$ label the branches, and let m_i be the number of HR events assigned to branch i, such that $\sum_{i=0}^{B} m_i = M$. For a given branch i, let $j = 1, ..., m_i$ label the recombination events, and let r_{ij} be the length of genetic tract, in DNA base pairs, replaced by the HR event.

We need to define branch lengths in the genealogy; the rates of recombination in our models are defined as rates per unit of branch length, and thus their interpretation will depend on the chosen measure of branch length. Since our model structure is generic with respect to this choice, we denote L_i^*, the generic length of branch i. In different analyses, we consider three different units of branch lengths. By default, the branch length is measured the length in years estimated using a dated genealogy based on a relaxed molecular clocked estimated using Bayesian methods, denoted L_i^{clock}. Alternatively, we also measure the branch length by the number of SNPs assigned to mutations along branch i, denoted L_i^{SNPs}, or by the length estimated using a substitution model in the maximum likelihood reconstruction of the genealogy, denoted L_i^{MLE}. The models we develop to fit to the data are expressed in terms of generic branch lengths, denoted L_i^*, where '$*$'$=\{\text{clock}, \text{SNPs}, \text{MLE}\}$.

Thus in summary, the data we aim to fit to are the number m_i and size r_{ij} of HR events given the branch lengths L_i^*.

2 General framework for inference

Generically, for a model X, we will have a set of n_p^X parameters, denoted $\Phi_X = \{\phi_a^X\}$, where $a = 1, ..., n_p^X$ label the parameters. The model is fitted by numerically maximizing the log likelihood

$$l_X(\Phi_X) \propto \ln\left(\prod_{i=1}^{B} f_X(m_i|\Phi_X, L_i^*) \prod_{j=1}^{m_i} s_X(r_{ij}|\Phi_X, L_i^*, m_i)\right) + \text{constant}, \tag{S1}$$

where as usual the likelihood is defined relative to an arbitrary constant (which we set, without loss of generality, to zero). f_X is the probability distribution of number of recombination events (the frequency distribution) and s_X is the probability distribution of size of recombination events conditional on their frequency (the size distribution). For models where the size of recombination events is independent of their frequency, so that $s_X(r_{ij}|\Phi_X, L_i^*, m_i)$ is

independent of m_i, the likelihood factorizes as

$$\begin{aligned} l_X(\Phi_X) &= l_X^{\text{freq}}\left(\Phi_X^{\text{freq}}\right) + l_X^{\text{size}}\left(\Phi_X^{\text{size}}\right) \\ l_X^{\text{freq}}\left(\Phi_X^{\text{freq}}\right) &= \ln\left(\prod_{i=1}^{B} f_X(m_i|\Phi_X^{\text{freq}}, L_i^*)\right) \\ l_X^{\text{size}}\left(\Phi_X^{\text{size}}\right) &= \ln\left(\prod_{i=1}^{B}\prod_{j=1}^{m_i} s_X(r_{ij}|\Phi_X^{\text{size}}, L_i^*)\right). \end{aligned} \tag{S2}$$

Above, Φ_X^{freq} is the subset of parameters which governs the frequency of recombination events and Φ_X^{size} is the subset of parameters which governs the size of recombination events, with $\Phi_X = \Phi_X^{\text{freq}} \cup \Phi_X^{\text{size}}$ and $\Phi_X^{\text{freq}} \cap \Phi_X^{\text{size}} = \varnothing$. In the case of factorisable models, the two likelihoods in equation (S2) can be maximised independently.

The models were compared by computing the adjusted Akaike's Information Criterion (AIC_c), defined as

$$AIC_c^X = -2l_X(\tilde{\Phi}_X) + 2n_p^X \frac{N_{DF}}{N_{DF} - n_p^X - 1} + \text{const}, \tag{S3}$$

where $\tilde{\Phi}_X$ is the set of maximum likelihood parameters for model X, n_p^X is the number of parameters for model X, and N_{DF} is the number of degrees of freedom, here given by

$$N_{DF} = B + \sum_{i=1}^{B} \max(m_i - 1, 0). \tag{S4}$$

A measure of the quality of fit relative to the best fitting model, defined as $AIC_{\text{ref}} = \min_X(AIC_c^X)$, was given by

$$\Delta AIC_c^X = AIC_c^X - AIC_{\text{ref}}. \tag{S5}$$

3 Standard definitions for probability distributions

Since we refer to these repeatedly, we denote $\text{Pois}(x|\alpha)$ the Poisson distribution of variable x with mean α, $\text{Geom}(x|\alpha)$ the geometric distribution of variable x with mean α and $\text{NegBin}(x|\alpha, k)$ the negative binomial distribution of variable x with mean α and dispersion coefficient k,

$$\begin{aligned} \text{Pois}(x|\alpha) &= \frac{\alpha^x e^{-\alpha}}{\Gamma(x+1)} \\ \text{Geom}(x|\alpha) &= \frac{1}{1+\alpha}\left(\frac{\alpha}{1+\alpha}\right)^x \\ \text{NegBin}(x|\alpha, k) &= \frac{\Gamma(k+x)}{\Gamma(k)\Gamma(x+1)} \frac{k^k \alpha^x}{(k+\alpha)^{k+x}}, \end{aligned} \tag{S6}$$

where $\Gamma(x)$ is a standard gamma function. The variance of the Poisson distribution is α, the variance of the geometric distribution is $\alpha(1+\alpha)$, and the variance of the negative binomial distribution is $\alpha(k+\alpha)/k$, a monotonically decreasing function of k. The negative binomial distribution is identical to the geometric distribution when $k = 1$ and becomes identical to the Poisson distribution in the limit $k \to \infty$. For the sake of later notational convenience, we define $\text{Pois}(0|0) = 1$ and $\text{Pois}(x|0) = 0$ for $x > 0$, and similarly $\text{NegBin}(0|0, k) = 1$ for any k and $\text{NegBin}(x|0, k) = 0$ for any k and for $x > 0$.

4 Defining the models

We now proceed to define the various models that we fit to the data.

Model 1: A null model of recombination

Our null model assumes that the rate of recombination events is Poisson distributed and the size distribution of recombination events is geometrically distributed. This stems from the (null) assumption that when a strand of DNA is transported into the cytoplasm during competence, there is a constant probability $1/(1+\Sigma)$ that the DNA of tract length Σ may be cut at any base, and a constant probability $\lambda\delta L$ that the DNA segment integrates into the cell's DNA (where L is time measured in units of phylogenetic distance). In other words, the rate of recombination is λ, so $\Phi_1^{\text{freq}} = \{\lambda\}$, and the mean size of recombinational replacements is Σ, so $\Phi_1^{\text{size}} = \{\Sigma\}$, and the probability functions that define the likelihood are:

$$\begin{aligned} f_1(m_i|\Phi_1^{\text{size}}, L_i^*) &= \text{Pois}(m_i|\lambda L_i^*) \\ s_1(r_{ij}|\Phi_1^{\text{freq}}, L_i^*) &= \text{Geom}(r_{ij}|\Sigma). \end{aligned} \tag{S7}$$

Model 2: An empirical extension of the null model with over-dispersion

This model is similar to model 1, but the Poisson and geometric distributions are replaced by more flexible negative binomial distributions, with dispersion parameters k_λ and k_Σ for rate and size, respectively, so that $\Phi_2^{\text{freq}} = \{\lambda, k_\lambda\}$ and $\Phi_2^{\text{size}} = \{\Sigma, k_\Sigma\}$, and

$$\begin{aligned} f_2(m_i|\Phi_2^{\text{size}}, L_i^*) &= \text{NegBin}(m_i|\lambda L_i^*, k_\lambda) \\ s_2(r_{ij}|\Phi_2^{\text{freq}}, L_i^*) &= \text{NegBin}(r_{ij}|\Sigma, k_\Sigma). \end{aligned} \tag{S8}$$

Model 3: A mixture model of micro- and macro-recombination

In this model, micro-recombination occurs at a rate λ, results in a recombinational event of mean size Σ. Macro-events occur at rate ρ, and result in mean Q simultaneous recombination events of mean size Ω. Unlike models 1 and 2, this model does not factorise, i.e. the size distribution is not independent of the frequency distribution. The parameter set for this model is $\Phi_3 = \{\lambda, \Sigma, \rho, Q, \Omega\}$.

In this model, the frequency distribution of recombination events is a combination of micro-recombination and macro-recombination, namely

$$f_3(m_i) = \sum_{s=0}^{m_i} p_{\text{micro}}(s)\; p_{\text{macro}}(m_i - s), \tag{S9}$$

where s is a random variable for the number of micro-recombinations and $m_i - s$ a random variable for the number of macro-recombinations. We know that s is Poisson distributed with mean λL_i^*, hence

$$p_{\text{micro}}(s) = \text{Pois}(s_i|\lambda L_i^*).$$

However, as macro-recombination gives rise to multiple, simultaneous recombination segments, the likelihood of $m_i - s$ recombinations through macro-recombination is a likelihood of $m_i - s$ recombinations given h macro-events, namely

$$p_{\text{macro}}(s) = \sum_{h=0}^{\infty} p(m_i - s|h)\; p(h).$$

As we assume that each macro-event happens at rate ρ, thus h is Poisson distributed with mean ρL_i^*. It can also be shown that if a single macro-event gives rise to, on average, Q recombinations (Poisson distributed), then $m_i - s$ will also be Poisson distributed with mean hQ as h events give rise to hQ recombinations. For example, for $h = 2$ we have

$$\begin{aligned} p(m_i - s|h = 2) &= \sum_{e=0}^{m_i - s} \text{Pois}(e|Q)\text{Pois}(m_i - s - e|Q) \\ &= \text{Pois}(m_i - s|2Q), \end{aligned}$$

and so on. Given all this, the frequency distribution of recombination events (S9) becomes

$$\begin{aligned} f_3(m_i|\Phi_3, L_i^*) &= \sum_{s_i=0}^{m_i} \text{Pois}(s_i|\lambda L_i^*) \sum_{h=0}^{\infty} \text{Pois}(h|\rho L_i^*)\,\text{Pois}(m_i - s_i|hQ) \\ &= \sum_{h=0}^{\infty} \text{Pois}(h|\rho L_i^*)\,\text{Pois}(m_i|\lambda L_i^* + hQ). \end{aligned} \tag{S10}$$

To simplify equation (S10) we have made extensive use of the fact that the convolution of two Poisson distributions is also a Poisson distribution, i.e.

$$\sum_{a=0}^{b} \text{Pois}(a|A_1)\,\text{Pois}(b - a|A_2) = \text{Pois}(b|A_1 + A_2).$$

To determine the size distribution of events, we need to determine the probability of there being s_i micro-recombination events and $m_i - s_i$ macro-recombination events along branch i. This is given by

$$p_3(s_i|\Phi_3, L_i^*, m_i) = \frac{\text{Pois}(s_i|\lambda L_i^*) \sum_{h=0}^{\infty} \text{Pois}(h|\rho L_i^*)\,\text{Pois}(m_i - s_i|hQ)}{f_3(m_i|\Phi_3, L_i^*)}. \tag{S11}$$

The size distribution is then given by

$$s_3(r_{ij}|\Phi_3, L_i^*, m_i) = \sum_{s_i=0}^{m_i} p_3(s_i|\Phi_3, L_i^*, m_i)\left(\frac{s_i}{m_i}\mathrm{Geom}(r_{ij}|\Sigma) + \frac{m_i - s_i}{m_i}\mathrm{Geom}(r_{ij}|\Omega)\right). \quad \text{(S12)}$$

Given a set $\tilde{\Phi}_3$ of maximum likelihood estimates for the parameter set Φ_3, we can estimate the probability $p_3(ij \subset H|\tilde{\Phi}_3, L_i^*, m_i, r_{ij})$ that any observed recombination event, labeled ij, is the result of macro-recombination (rather than micro-recombination), and thus belongs to the set H of macro-recombination events

$$p_3(ij \subset H|\tilde{\Phi}_3, L_i^*, m_i, r_{ij}) = \frac{\sum_{s_i=0}^{m_i} p_3(s_i|\tilde{\Phi}_3, L_i^*, m_i)\frac{m_i - s_i}{m_i}\mathrm{Geom}(r_{ij}|\tilde{\Omega})}{s_3(r_{ij}|\tilde{\Phi}_3, L_i^*, m_i)}. \quad \text{(S13)}$$

Model 4: An adapted version of the mixture model with similar heterogeneities but no link between frequency and size of recombination events

In the mixture model of micro- and macro-recombination, model 3 above, both the rate of events and the size of events arise as mixtures, and these are linked by virtue of the fact that the macro-recombination process is assumed to give rise to both a larger number and bigger recombination events. To test whether this link is supported by the data, we consider a model which is identical in every way to model 3 except for the absence of this link. To do this, we need one extra parameter, denoted σ, the probability that any given recombination event is small (with mean size Σ) or large (with mean size Ω). Unlike model 3, this model factorizes into a model for frequencies and a model for sizes, with parameter sets $\Phi_4^{\mathrm{freq}} = \{\lambda, Q, \rho\}$ and $\Phi_4^{\mathrm{size}} = \{\sigma, \Sigma, \Omega\}$. The probability functions for frequency and size are

$$\begin{aligned} f_4(m_i|\Phi_4^{\mathrm{size}}, L_i^*) &= \sum_{h=0}^{\infty} \mathrm{Pois}(h|\rho L_i^*)\mathrm{Pois}(m_i|\lambda L_i^* + hQ) \\ s_4(r_{ij}|\Phi_4^{\mathrm{freq}}, L_i^*) &= (1-\sigma)\,\mathrm{Geom}(r_{ij}|\Sigma) + \sigma\,\mathrm{Geom}(r_{ij}|\Omega). \end{aligned} \quad \text{(S14)}$$

This model becomes equivalent to the mixture model 3 if the parameter σ is replaced by the function of m_i, $\sum_{s_i=0}^{m_i} p_3(s_i|\tilde{\Phi}_3, L_i^*, m_i)\frac{m_i - s_i}{m_i}$.

5 Numerical implementation

The likelihoods are coded algebraically in Mathematica 8.0, and for each model and for each choice of branch length metrics, the likelihood is numerically maximized with respect to full parameter sets using Mathematica's global optimization function `NMaximize` or `FindMaxiumum` with default settings. Confidence intervals are estimated using numerically determined univariate likelihood profiles. Several of the probabilities require sums over infinite series of possible macro-recombination events, of the form: $\sum_{h=0}^{\infty}$, such as in equations (S11), and (S14). To speed up the numerical estimation of these sums, we approximate these sums by finite sums of the form: $\sum_{h=0}^{h_{\max}}$ where $h_{\max}$ is a suitably large integer. Suitability is determined by

convergence of the maximum likelihood estimates of parameter sets as a function of h_{max}. In practice we find that $h_{max} = 30$ is more than suitable, and permits rapid numerical estimation of the relevant likelihoods.

6 Goodness of fit

Goodness of fit is determined verifying the ability of the model to replicate the data under re-simulation. For a given model X, a given set of maximum likelihood parameters $\tilde{\Phi}_X$, and a the observed set of branch lengths $\{L_i^*\}$, many simulated sets of recombination events is generated by simulation. Marginal distributions (of frequency per branch, and size) of the simulations are compared to the equivalent marginal distributions of the data (see Figures 3 and 4).
