# Supplementary material for: Heterogeneity in the Frequency and Characteristics of Homologous Recombination in Pneumococcal Evolution
Source: PLoS Genet. 2014 May 1;10(5):e1004300. doi: 10.1371/journal.pgen.1004300 (PMC4006708; doi:10.1371/journal.pgen.1004300)
Supplement: Text S2 — Additional results. Figures S1, S2, S3, S4, S5,S6 and Tables S1, S2 and S5, S6, S7, S8. (PDF) [file pgen.1004300.s016.pdf]

Text S2. Additional results.

Summary

Results for alternative branch lengths

In addition to the branch lengths measured in years estimated using a relaxed molecular clock, here we present the results for two additional measures of branch lengths (see also Text S1). The first measure (MLE) is a substitution model from the maximum likelihood reconstruction of the genealogy. The second measure (SNPs) is based on the number of SNPs assigned to mutations along each branch. The results of the model fitting for PMEN1 are given in Table S1, and the results of the model fitting for CC180 are given in Table S2. The best fit of the model to data for both measures of branch length is shown for PMEN1 in Fig. S1 and for CC180 in Fig. S2. These show that the results are robust with respect to the branch length used.

Saturation of the mismatch repair

One of the hypotheses for the observation of micro- and macro-recombination, as discussed in the main text, is the saturation of the mismatch repair (MMR). To address this hypothesis, we plotted the distribution of the number of SNPs at micro-branches versus the distribution of the number of SNPs within macro-branches (see Figures S5 and S6). We used two different ways of defining micro-/macro-branches. According to the 'all or none' definition, a branch was named a micro-branch (macro-branch) if all events on this branch were micro-recombinations (macro-recombinations). According to the 'majority' definition, a branch was named a micro-branch (macro-branch) if the majority events on this branch were micro-recombinations (macro-recombinations).

An alternative test used was to quantify the type of mutations within micro- and macro-recombinations associated with the efficiency of the MMR. In particular, certain substitutions (so called 'low-efficiency markers') are known to be repaired most efficiently by MMR and some others ('high-efficiency markers') to be repaired less efficiently by MMR. To test for a difference in distribution of the efficiency of markers between micro- and macro-recombination (where macro-recombination was defined as of posterior probability $p \geqslant 75\%$), we performed a chi-square test on the measured numbers, summarised in Table S5. We observe a significant association in both PMEN1 and CC180, even though the magnitude of the effect is weak in PMEN1. Interestingly, however, we did not find any association in simulations with a preassumed mixture model, defined as model C in Text S3.

Heterogeneity of recombinations at non-surface loci

As explained in the main text, one hypothesis for the observed heterogeneity could be the selective pressure acting on major surface antigens. Tables S6 and S7 show results of the models fitted to data of PMEN1 and CC180, respectively, with recombinations affecting those loci removed. After the removal, the mixture model 3 remains the best fit in both cases.

Effects of over-sampling and vaccine-induced selective pressure

To address the concern that the observed heterogeneity is the result of the non-systematic nature of the isolate collections used in the original analysis of PMEN1, we removed two groups of isolates from the data. First, we removed the all branches upstream from samples collected in South Africa due to extensive sampling in that region. Second, we removed all isolates classified as 19A to rule out potential PCV7 vaccine-induced selective pressure. The results are shown in Table S8. One can see that the fit is qualitatively identical to the one obtained based on the full dataset. Thus, we're confidence that neither over-sampling nor vaccine are the reason for the observed heterogeneity.

Alternative presentation of the distribution of homologous recombinations

The distribution of recombination events in Fig. 5 in main text explains well how recombination events map on the clonal phylogeny of each lineage. However, this makes difficult to envisage how all inferred recombination events distribute along the genome. To visualise this, we plotted all inferred recombination events branch-by-branch, sorted by the branch length. The distribution for PMEN1 can be found in Fig. S3 and for CC180 can be found in Fig. S4.

A: MLE

**Model**	AIC_c	ΔAIC_c	λ	Σ	k_λ	k_Σ	ρ	Ω	Q	σ
1 (NM)	13,295	479	1300	6,400	–	–	–	–	–	–
2 (NMOD)	12,909	93	1300	6,400	1.4	0.53	–	–	–	–
3 (MM)	12,816	0	390	550	–	–	490	8,900	1.9	–
4 (UMM)	12,850	34	840	160	–	–	90	7,800	5.3	0.83

B: SNPs

**Model**	AIC_c	ΔAIC_c	λ	Σ	k_λ	k_Σ	ρ	Ω	Q	σ
1 (NM)	13,441	516	0.088	6,400	–	–	–	–	–	–
2 (NMOD)	12,966	41	0.095	6,400	0.99	0.53	–	–	–	–
3 (MM)	12,925	0	0.026	580	–	–	0.028	8,900	2.2	–
4 (UMM)	12,960	35	0.052	150	–	–	0.0066	7,700	5.4	0.83

Table S1. Model comparison of four models for the PMEN1 tree with two remaining units of branch lengths. (A) Branch length is estimated using a substitution model in the maximum likelihood reconstruction of the genealogy. (B) Branch length is measured by the number of SNPs assigned to mutations along branch. Data are displayed as in Table 1.

A: MLE

**Model**	AIC_c	ΔAIC_c	λ	Σ	k_λ	k_Σ	ρ	Ω	Q	σ
1 (NM)	1,973	329	70	11,000	–	–	–	–	–	–
2 (NMOD)	1,738	94	40	11,000	0.12	0.47	–	–	–	–
3 (MM)	1,644	0	10	27	–	–	4.7	14,000	13	–
4 (UMM)	1,699	55	17	26	–	–	3.5	14,000	15	0.82

B: SNPs

**Model**	AIC_c	ΔAIC_c	λ	Σ	k_λ	k_Σ	ρ	Ω	Q	σ
1 (NM)	1,878	232	0.023	11,000	–	–	–	–	–	–
2 (NMOD)	1,731	84	0.011	11,000	0.22	0.47	–	–	–	–
3 (MM)	1,646	0	0.0034	27	–	–	0.0024	14,000	8	–
4 (UMM)	1,690	44	0.0057	26	–	–	0.0012	14,000	15	0.82

Table S2. Model comparison of four models for the CC180 tree with two remaining units of branch lengths. (A) Branch length is estimated using a substitution model in the maximum likelihood reconstruction of the genealogy. (B) Branch length is measured by the number of SNPs assigned to mutations along branch. Data are displayed as in Table 1.

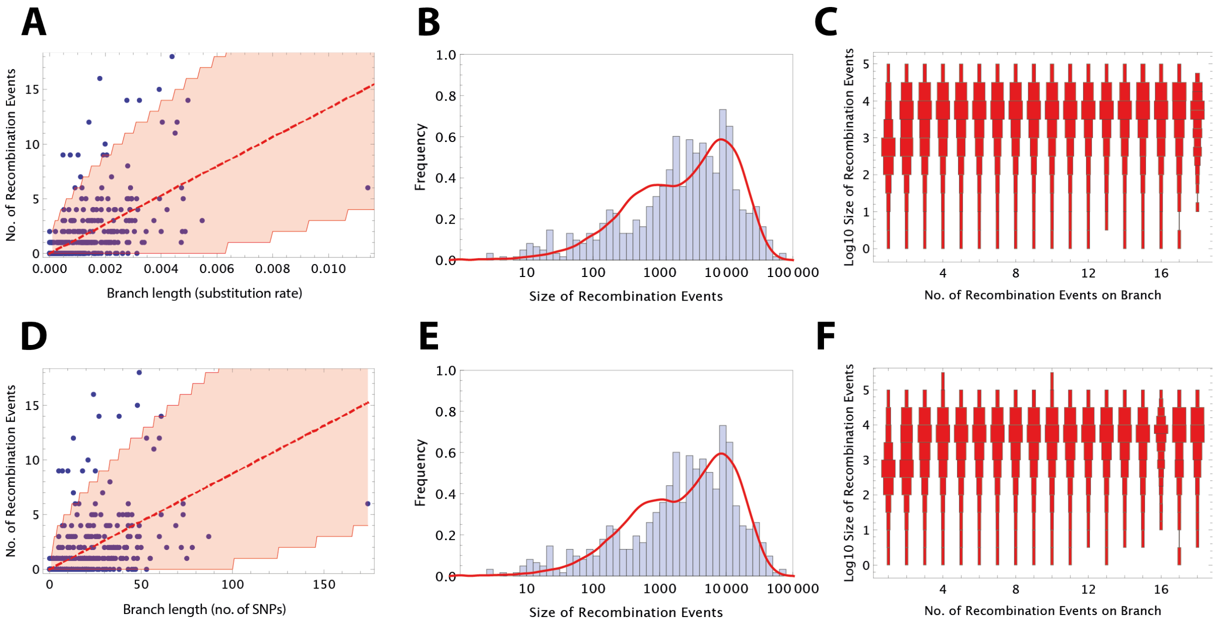

Figure S1. Goodness of fit of the best-fitting mixture model 3 for two alternative branch length units (PMEN1). (A-C) Results for the underlying tree with branch lengths as substitution rates of the maximum likelihood estimate. (D-F) Results for the underlying tree with branch lengths as numbers of SNPs. Data are displayed as in Fig. 3G-H.

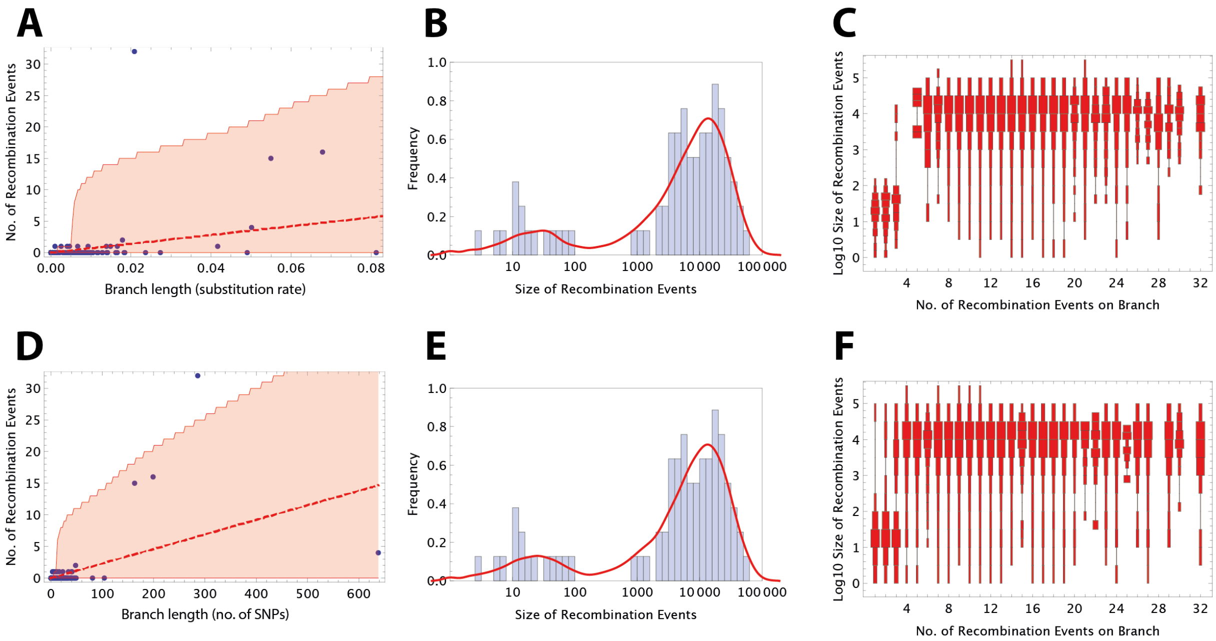

Figure S2. Goodness of fit of the best-fitting mixture model 3 for two alternative branch length units (CC180). (A-C) Results for the underlying tree with branch lengths as substitution rates of the maximum likelihood estimate. (D-F) Results for the underlying tree with branch lengths as numbers of SNPs. Data are displayed as in Fig. 3G-H.

PMEN1 ($p = 0.001$)

		type of substitution		
		low	mid	high
**type of rec.**	micro	1,235	159	418
	macro	28,466	3,376	7,778

CC180 ($p = 2.36 \times 10^{-10}$)

		type of substitution		
		low	mid	high
**type of rec.**	micro	29	15	29
	macro	6,666	644	1,780

Simulation: run 1 ($p = 0.3$)

		type of substitution		
		low	mid	high
**type of rec.**	micro	137	9	37
	macro	18,391	2,034	4,865

Simulation: run 2 ($p = 0.39$)

		type of substitution		
		low	mid	high
**type of rec.**	micro	251	24	63
	macro	16,695	2,040	4,754

Simulation: run 3 ($p = 0.94$)

		type of substitution		
		low	mid	high
**type of rec.**	micro	194	23	52
	macro	15,407	1,932	4,301

Table S5. Heterogeneity of recombination versus 'marker efficiency'. Markers were subdivided according to three types of substitutions considered: low-efficiency markers (transitions), mid-efficiency markers (transversions $A/T \leftrightarrow T/A$), and high-efficiency markers (transversions $A/T \leftrightarrow C/G$ and $C/G \leftrightarrow G/C$). The lower the efficiency of a polymorphism, the higher the probability of being repaired by the MMR. In PMEN1 and CC180 we see a significant association between the two properties, namely macro-recombinations have more low-efficiency markers and less high-efficiency markers than expected from a random process. However, these associations are not observed in three simulations of micro- and macro-recombination.

**Model**	AIC_c	ΔAIC_c	λ	Σ	k_λ	k_Σ	ρ	Ω	Q	σ
1 (NM)	9,935	501	0.14	6,100	–	–	–	–	–	–
2 (NMOD)	9,493	68	0.15	6,100	0.47	0.59	–	–	–	–
3 (MM)	9,425	0	0.037	770	–	–	0.033	8,000	3.2	–
4 (UMM)	9,505	79	0.047	220	–	–	0.026	7,200	3.7	0.84

Table S6. Model comparison of four models for recombinations occurring outside of five major antigen loci in PMEN1 (pspA, cps, pclA, psrP and pspC). Recombination events were removed when they fully spanned any of the loci, when they occurred within any of the loci or when they partially overlapped with any of the loci. The number of degrees of freedom in the data is $N_{DF} = 778$. The layout of the table is identical to the one in Tables 1 and 2 in main text.

Model	AIC_c	ΔAIC_c	λ	Σ	k_λ	k_Σ	ρ	Ω	Q	σ
1 (NM)	1,873	321	0.018	10,000	–	–	–	–	–	–
2 (NMOD)	1,643	92	0.011	10,000	0.11	0.46	–	–	–	–
3 (MM)	1,552	0	0.0030	27	–	–	0.0012	13,000	12	–
4 (UMM)	1,607	55	0.0044	26	–	–	0.00097	13,000	14	0.81

Table S7. Model comparison of four models for recombinations occurring outside of three major antigen loci in CC180 (pspA, cps and pspC) in analogy to Table S6. The number of degrees of freedom in the data is $N_{DF} = 222$.

A: African samples excluded

Model	AIC_c	ΔAIC_c	λ	Σ	k_λ	k_Σ	ρ	Ω	Q	σ
1 (NM)	10,820	435	0.18	6,600	–	–	–	–	–	–
2 (NMOD)	10,429	45	0.19	6,600	1.0	0.52	–	–	–	–
3 (MM)	10,384	0	0.061	680	–	–	0.050	9,400	2.4	–
4 (UMM)	10,437	53	0.11	270	–	–	0.012	8,200	6.0	0.78

B: 19a serotypes excluded

Model	AIC_c	ΔAIC_c	λ	Σ	k_λ	k_Σ	ρ	Ω	Q	σ
1 (NM)	12,472	491	0.19	6,300	–	–	–	–	–	–
2 (NMOD)	12,011	30	0.20	6,300	0.93	0.53	–	–	–	–
3 (MM)	11,980	0	0.059	600	–	–	0.058	8,800	2.3	–
4 (UMM)	12,010	30	0.12	150	–	–	0.012	7,700	6.1	0.82

Table S8. Do isolate over-sampling or vaccine have any impact on the inference of heterogeneity? Two subdatasets were generated: (A) subset of data based on samples which did not come from Africa, and (B) subset of data based on samples which were not serotyped as 19A.

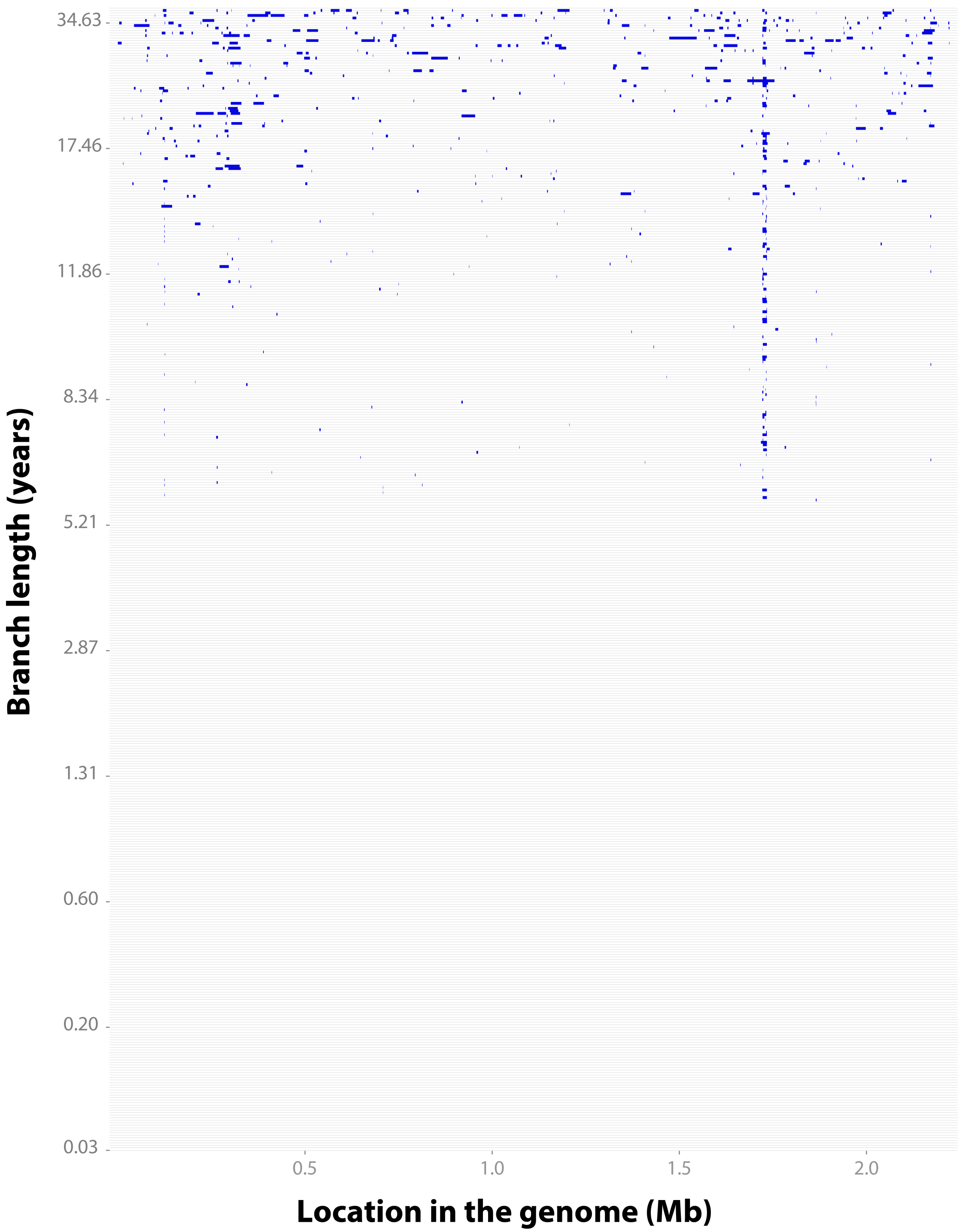

Figure S3. Distribution of recombinations for PMEN1. Each horizontal line represents a full genome at each branch of the tree, and the blue squares correspond to the positions at which recombination events have been found. The lines are sorted according to the inferred branch length. Branches of the same length were plotted on a single line, with blue squares denoting positions at which recombinations have been detected at any of these branches. A single blue pixel corresponds to a window of the size 200 bp in which any recombination events have been detected.

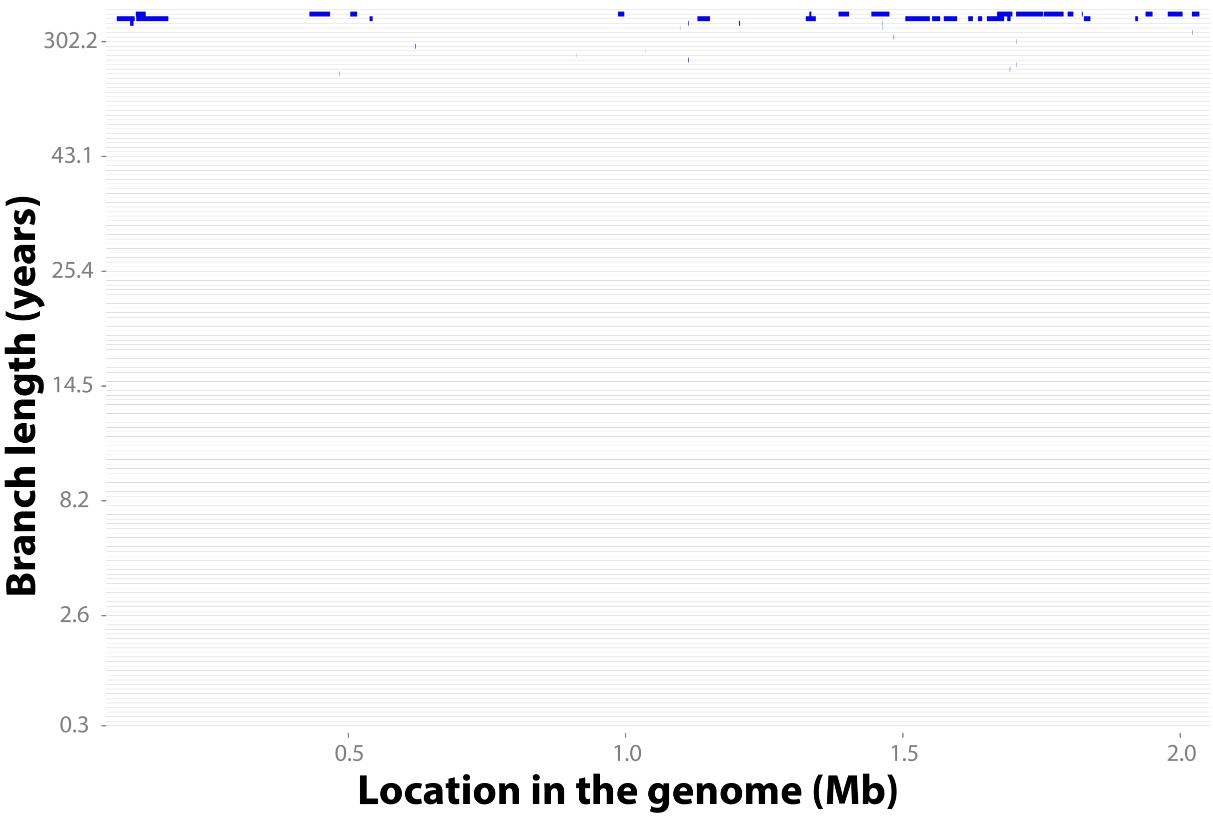

Figure S4. Distribution of recombinations for CC180. Data are displayed as in Fig. S3.

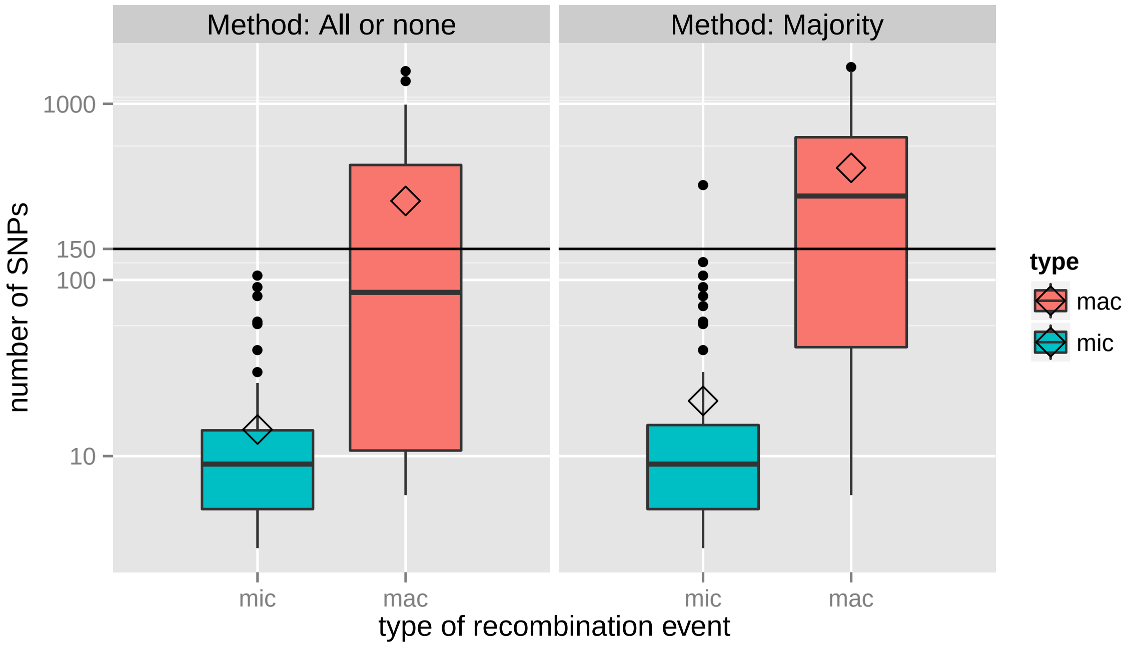

Figure S5. Micro/macro-recombination vs. saturation of the mismatch repair (MMR) in PMEN1. Box-plots show the distribution of the number of SNPs in branches on which micro-recombinations occur (green) and those on which macro-recombinations occur (red). Two methods to classify branches were used: based on all events on a given branch being of the same type (left), or based on the predominating type on a given branch (right); branches failing to fulfil either condition were not plotted. The square diagram show the mean value per box-plot. The number of SNPs hypothesised as a MMR saturation threshold (150) is plotted as a black horizontal line.

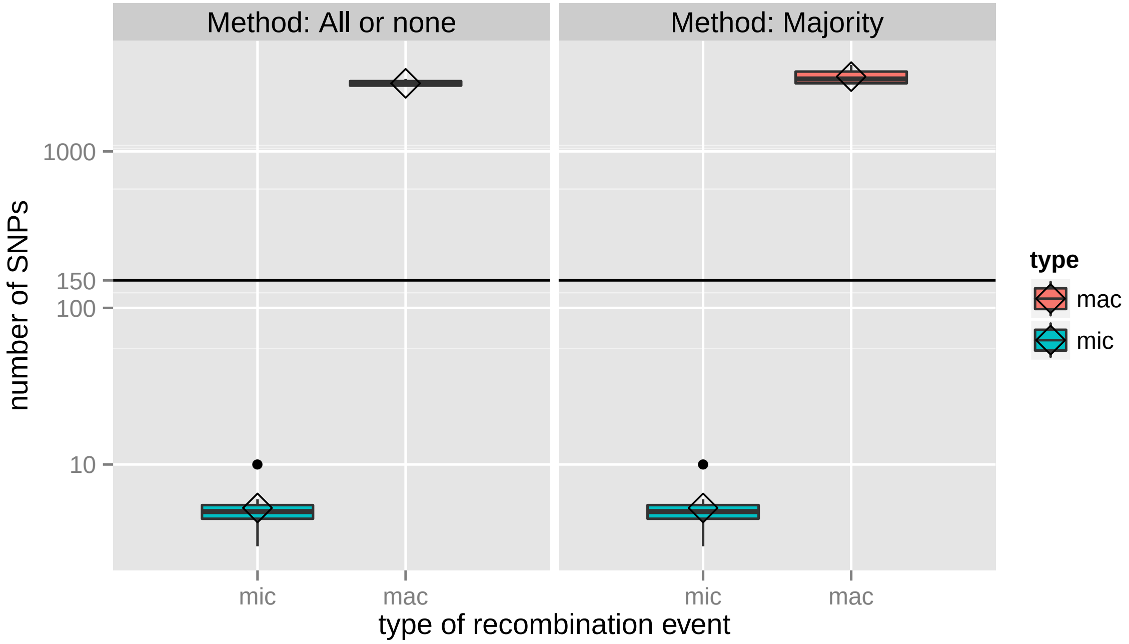

Figure S6. Micro/macro-recombination vs. saturation of the mismatch repair (MMR) in CC180. Data are displayed as in Figure S5.
