# Supplementary material for: Heterogeneity in the Frequency and Characteristics of Homologous Recombination in Pneumococcal Evolution
Source: PLoS Genet. 2014 May 1;10(5):e1004300. doi: 10.1371/journal.pgen.1004300 (PMC4006708; doi:10.1371/journal.pgen.1004300)
Supplement: Text S3 — Simulations of heterogeneity of recombination. Tables S3, S3 S4. (PDF) [file pgen.1004300.s017.pdf]

Text S3. Simulations of heterogeneity of recombination.

Here we simulate diversification of a set of *S. pneumoniae* through different models of recombination. We then examine whether the preassumed model of recombination can be correctly inferred from the simulated data. The summary of the fits is given in Table S3. The details of the simulation follow.

The ancestral genotype was taken as that of *S. pneumoniae* ATCC 700669 [EMBL accession code: FM211187], the earliest known isolate of the PMEN1 lineage. This lineage served as an ancestor of all simulated sequences. Diversification was simulated from $t = 0$ to $t = t_{max}$ in discrete steps. At each step, every extant sequence acquired a single base substitution. Furthermore, each sequence had a probability p_C of being duplicated into two independently diversifying sequences, corresponding to a coalescent event in the phylogeny. Each sequence also had a probability p_S of being sampled at each timestep, after which point it no longer diversified or served as the progenitor to any other sequences. The simulation was stopped at t_{max} once the total number of sampled and extant sequences in the simulation reached (or exceeded) a pre-determined maximum, n_{max}.

At each discrete time step, recombinations occurred with a fixed probability p_R depending on the preassumed model (see below). These involved exchanging the relevant region of the recipient sequence in the alignment for the corresponding homologous sequence from a randomly-selected donor. The list of donor sequences consisted of 18 publicly available whole genomes of *S. pneumoniae*, and is shown in Table S4. For each of the donor sequences was mapped against a reference and aligned using the following pipeline. One hundred nucleotide paired end Illumina reads, with an insert size of 250 bp were simulated from each of the donor sequences. These were then mapped against the *S. pneumoniae* ATCC 700669 genome using SMALT v0.7.8 and bases called using the same criteria as described in [1] to replicate the process by which the original whole genome alignment of PMEN1 isolates was generated. This was used to generate a whole genome alignment that served as the input for simulations of diversification through different recombination processes. Recombination events had a random start location, and extended for a length following a geometric distribution (defined by a λ parameter) also dependent on the preassumed model A, B, C or D.

Model A: Regular recombination model. Under this model, recombinations occurred with $p_R = 0.1$. Their lengths followed a geometric distribution described by the parameter λ_R values of 0.00016 bp^{-1} (corresponding to a mean length of 6.3 kb). These parameter estimates are taken from the original analysis of the PMEN1 lineage.

Model B: Heterogeneous recombination model. Under this model, two modes of recombination occur. Both micro-and macro-recombinations independently occurred with the same $p_R = 0.03$, with the size distributions of both defined by λ_R. Each instance of micro-recombination introduced a single event whereas each instance of macro-recombination introduced Q segments of sequence, where Q was randomly drawn from a Poisson distribution with a mean of 2.3. These values were taken from the fit of the mixture model to the PMEN1 data described in this study.

Model C: Correlated mixture model. Under this model, the micro- and macro-recombinations both again occurred independently with the same $p_R = 0.03$. However, in this case the different processes were associated with different length distributions: micro-recombinations were defined using $\lambda_\Sigma = 0.0021$ bp^{-1} (corresponding to a mean length of 480 bp), whereas macro-recombinations were defined by $\lambda_\Omega = 0.00011$ bp^{-1} (corresponding to a mean length of 8.8 kb). These values were taken from the fit of the mixture model to the PMEN1 data described in this study.

Model D: Uncorrelated mixture model. Under this model, the micro- and macro-recombinations both again occurred independently with the same $p_R = 0.03$. Each instance of micro-recombination

introduced a single event whereas each instance of macro-recombination introduced Q segments of sequence, where Q was randomly drawn from a Poisson distribution with a mean of 2.3. However, both micro- and macro-recombinations were an equal mix of events drawn from the two size distributions defined by $\lambda_\Sigma = 0.0021$ bp^{-1} and $\lambda_\Omega = 0.00011$ bp^{-1}.

Three simulations were run for each of the models, all of which used $n_{max} = 242$ generated with the parameter values $p_C = 0.05$ and $p_S = 0.025$. The alignment of each set of sequences was then analysed as described for the original set of PMEN1 isolates. The different models of recombination were then fitted to these outputs, the results of which are displayed in Table S3. Each of the preassumed models A-D was correctly identified by fitting the correct model to the data.

- Model A had a homogeneous distribution of frequency and size of recombination, and indeed the homogeneous model 1 turned out to be the best fit.
- Model B had a heterogeneous distribution of frequency but not size. Indeed, in all three cases the mixture model 3 was the best fit to the data. In 2/3 runs, the inferred sizes were correctly identified as equal, whereas in 1/3 runs they were inferred as comparable.
- Model C had a heterogeneous distribution of both frequency and size, making it very similar to the hypothesised mixture model 3. As expected, the mixture model 3 was by far the best fit to the data in each of the three runs.
- Model D was a variation of model C with independency between frequency and size, in similarity to the uncorrelated mixture model 4. In 2/3 runs there was no significant difference in the fit of model 3 and model 4, making the mixture model 3 a better fit by the rule of maximal parsimony. However, in one run model 4 was a very strong fit to the simulated data. This suggests that the model 4 is the best fit overall, with the expected caveat that stochastic effects may sometimes hinder the inference of the correct recombination model.

**Model**	**Run**	**ΔAIC$_c$ Model1**	**ΔAIC$_c$ Model 2**	**ΔAIC$_c$ Model 3**	**ΔAIC$_c$ Model 4**	**Mean micro**	**Mean macro**
A	1	**0**	3	6	9	5,200	9,200
A	2	**0**	1	DNC	8	NA	NA
A	3	**0**	2	DNC	9	NA	NA
B	1	119	28	**0**	1	5,900	5,900
B	2	120	21	**0**	2	5,500	5,500
B	3	125	26	**0**	4	4,900	6,800
C	1	256	61	**0**	34	570	8,000
C	2	250	46	**0**	43	960	8,400
C	3	184	46	**0**	19	680	9,400
D	1	69	11	**6**	0	400	8,000
D	2	115	42	**0**	9	620	9,100
D	3	78	33	39	**0**	6,100	6,100

Table S3. Results of model fitting to simulated data. DNC = did not converge.

Genome	Accession code
Streptococcus pneumoniae 670-6B	CP002176
Streptococcus pneumoniae 70585	CP000918
Streptococcus pneumoniae AP200	CP002121
Streptococcus pneumoniae CGSP14	CP001033
Streptococcus pneumoniae D39	CP000410
Streptococcus pneumoniae G54	CP001015
Streptococcus pneumoniae gamPNI0373	CP001845
Streptococcus pneumoniae Hungary19A-6	CP000936
Streptococcus pneumoniae INV104	FQ312030
Streptococcus pneumoniae INV200	FQ312029
Streptococcus pneumoniae JJA	CP000919
Streptococcus pneumoniae OXC141	FQ312027
Streptococcus pneumoniae P1031	CP000920
Streptococcus pneumoniae R6	AE007317
Streptococcus pneumoniae ST556	CP003357
Streptococcus pneumoniae Taiwan19F-14	CP000921
Streptococcus pneumoniae TCH8431/19A	CP001993
Streptococcus pneumoniae TIGR4	AE005672

Table S4. Details of sequences used as sequence donors in simulations.

References

[1] Croucher NJ, Harris SR, Barquist L, Parkhill J, Bentley SD (2012) A high-resolution view of genome-wide pneumococcal transformation. PLoS Pathog 8: e1002745.
